# Characterizing the dynamics of multi-scale global high impact weather events

**DOI:** 10.1038/s41598-024-67662-x

**Published:** 2024-08-15

**Authors:** Lawrence R. Frank, Vitaly L. Galinsky, Zhenhai Zhang, F. Martin Ralph

**Affiliations:** 1 Center for Scientific Computation in Imaging, University of California at San Diego, La Jolla, CA 92037-0854 USA; 2 Center for Western Weather and Water Extremes, University of California at San Diego, La Jolla, CA 92093-0854 USA

**Keywords:** Climate and Earth system modelling, Atmospheric dynamics

## Abstract

The quantitative characterization and prediction of localized severe weather events that emerge as coherences generated by the highly non-linear interacting multivariate dynamics of global weather systems poses a significant challenge whose solution is increasingly important in the face of climate change where weather extremes are on the rise. As weather measurement systems (multiband satellite, radar, etc) continue to dramatically improve, increasingly complex time-dependent multivariate 3D datasets offer the potential to inform such problems but pose an increasingly daunting computational challenge. Here we describe the application to global weather systems of a novel computational method called the Entropy Field Decomposition (EFD) capable of efficiently characterizing coherent spatiotemporal structures in non-linear multivariate interacting physical systems. Using the EFD derived system configurations, we demonstrate the application of a second novel computational method called Space-Time Information Trajectories (STITs) that reveal how spatiotemporal coherences are dynamically connected. The method is demonstrated on the specific phenomenon known as atmospheric rivers (ARs) which are a prime example of a highly coherent, in both space and time, severe weather phenomenon whose generation and persistence are influenced by weather dynamics on a wide range of spatial and temporal scales. The EFD reveals how the interacting wind vector field and humidity scalar field couple to produce ARs, while the resulting STITS reveal the linkage between ARs and large-scale planetary circulations. The focus on ARs is also motivated by their devastating social and economic effects that have made them the subject of increasing scientific investigation to which the EFD may offer new insights. The application of EFD and STITs to the broader range of severe weather events is discussed.

## Introduction

Understanding and quantifying the interrelated complexities of the dynamical influences of Earth’s weather patterns that occur on a wide range of spatial and temporal scales remains an ongoing challenge to meteorology. It is not an understatement that the spectre of climate change has raised the stakes in this quest because of the well-known inherent difficulties in predicting the dynamics of highly non-linear multi-scale multi-variate coupled systems. A major concern is the emergence of increased extreme weather events^1,2^ and their associated global ecological and socioeconomical costs^3–6^. This includes the influence on severe weather, such as thunderstorms^7,8^, which occur on the mesoscale, and atmospheric rivers (ARs), which are synoptic scale phenomena^9^. The development of methods for understanding the dynamics and evolution of severe weather events is therefore of ever increasing importance. The ever increasing sophistication and capabilities of weather measurement systems (multiband satellite, radar, etc) provides increasingly complex time-dependent multivariate 3D datasets offer the potential to inform such problems but pose an increasingly daunting computational challenge. In this paper we describe the application to global weather systems of a novel computational method called the Entropy Field Decomposition (EFD) capable of efficiently characterizing coherent spatiotemporal structures in non-linear multivariate interacting physical systems. While the method is quite general, its capabilities are best highlighted by application to a specific phenomenon of significant societal impact. ARs are a natural choice not only because of the devastating ecological and socioeconomic impacts^10^ but also because they are highly localized in both their spatial extent (synoptic) and their temporal evolution (days), and yet are influence by a wide range of physical effects at multiple scales, from planetary to mesoscale. Increasing attention is being paid to the development of computational methods for their genesis, development and termination^11,12^

Atmospheric rivers (**ARs**) are long, narrow, and transient corridors of strong water vapor transport. They play an important role in precipitation in many regions globally, such as the West Coast of U.S. where they are the main mechanism to advect moisture^13–16^. ARs contribute up to 50% of the annual precipitation over the western U.S.^17,18^, which is critical to the water supply. Meanwhile, ARs cause a majority of the major floods over the same region^15,19^. Due to the significant social and economic impacts, there has been growing interests and demands in scientific understanding and operational predicting of ARs^16,20^. Enhancing our ability to detect, characterize and predict ARs would therefore be of significant societal impact. Complicating this task is the fact that ARs are affected by interacting physical processes on multiple scales, from planetary-scale, synoptic-scale, to mesoscale, such as Rossby wave breaking, extratropical cyclones, low-level jets, and mesoscale frontal waves^21–24^. Characterizing the interactions of these multiscale non-linear phenomena that have great impacts on ARs therefore poses a significant data analysis challenge.

There is a vast literature and growing on the study of ARs including a growing literature on their detection and identification (see, for example, the ARTMIP project^25–28^) including a variety of ways to investigate AR life cycles^29,30^. Nevertheless, the complexity of the non-linear processes responsible for ARs leaves room for new approaches to perhaps augment the existing literature on detection, characterization, and prediction of ARs.

In this paper we report our results of an initial investigation of the application of the recently developed *entropy field decomposition* (**EFD**)^31,32^ to this problem. The EFD is not just an AR detection method, it is a general methodology for estimating and ranking space-time modes of complex, interacting, non-linear systems from noisy data that has proven particularly useful where exceedingly complex interacting spatially and temporally varying non-linear fields from unique events preclude the use of linear methods or those that require similarity between events via training data. In addition, *space-time information trajectories* (**STIT**) constructed as highest probability pathways within the modes provide a quantitative measure of large scale connectivity. EFD’s use of highly relevant prior information embedded *within any individual dataset* facilitates its use in individual unique events. Such is the case in severe local storms where we have previously demonstrated the utility of the EFD approach^33^.

This paper presents the results of applying EFD to global weather patterns and the question of AR detection using three high-impact AR events. The first two considered, in March 2005 and January 2021, had significant environmental and financial impact on the U.S. West Coast. We also reconsider one of the most famous historical storms, the “Great Storm of 1987” that occurred over the UK on Oct 15-16, 1987. This storm is considered historically significant because of its rapid development and the subsequent failures to predict its location and intensity. While the wind damage has been discussed in great detail (see, for example^34^) what is less well known is that this storm also produced an exceptionally strong AR. This is readily confirmed by reanalysis of the precipitation data. The rapid formation of an intense localized AR is therefore an excellent test case study on which to test our EFD methodology.

We demonstrate two main results. First, the EFD modes generated from just the wind field coupled with the specific humidity field are able to accurately reveal and rank different fully 3D+t dynamical modes of the evolution of the ARs, where by “3D+t” we denote that all scalar or vector fields depend on three spatial variables (latitude, longitude, and height), as well as on the time variable *t*. Secondly, the STITs successfully capture the large-scale flow pattern, which is similar to Rossby Waves patterns, and reveal that ARs are coincident with the eastern edge of the troughs in the STITs, which is consistent with the troughs of Rossby Waves. These results suggest that EFD provides a unique novel data analysis method with the potential for better understanding of the generation, maintenance, and intensity of ARs.

## Background

The Earth’s atmosphere is a highly complex dynamical physical system that gives rise to a wide variety of coherent phenomena, including atmospheric waves such as Rossby and equatorial waves, and other coherent phenomena (e.g., vortices) that are associated with severe weather events (hurricanes, tornadoes, atmospheric rivers, etc). These coherent phenomena exist at a wide range of spatial and temporal scales and are generated by a multitude of non-linear processes. The problem of detecting and quantitatively characterizing such multi-scale coherences is a difficult problem in estimation theory, and is a very active area of research in the meteorological community because it is indeed a non-trivial problem. Recent work includes the identification of Rossby waves^35–38^, mesoscale eddies that “masquerade” as Rossby waves^39^, and various methods for the identification of equatorial waves (for a review, see^40^).

Estimation of coherent non-linear phenomena from data is not specific to meteorology, of course, but is an active area of research in a wide range of scientific disciplines. This ubiquitous need suggests the importance of the development of general frameworks for analyzing time-varying volumetric data from observations. This was the motivation for the development of the EFD framework, which was developed from first principles from the combination of Bayesian probability theory and the physics discipline of field theory. A brief overview of this logical development of EFD is presented in Appendix A. This includes a brief discussion of inverse problems in meteorology (i.e., characterizing physical systems from data), how to formulate a general probabilistic framework from Bayesian theory, and the key role of prior information in the identification of high-probability solutions to the inverse problem. Finally, the concept of identifying extended coherent structures in both space and time using space-time information trajectories (STITs) is shown. This Appendix highlights how EFD and STIT analysis provide a novel automated yet formally consistent method for quantitatively characterizing these multiscale coherences.

Analyzing data from highly non-linear dynamical systems is a ubiquitous problem throughout a broad range of scientific disciplines, but perhaps best known in meteorology as a consequence of the pioneering work of Edward Lorenz^41–43^ who recognized the difficulty such complex physical systems posed for prediction. This led him to the formulation of *empirical orthogonal functions* (**EOF**)^44^ designed as a ’model free’ approach to detecting the primary spatiotemporal patterns, or ’modes’, of weather systems. As discussed in greater detail in Appendix A, the EOF has some significant limitations, but the spirit of the approach is correct: How does one extract the most probable modes of a complex system from the data with minimal assumptions? It was from this viewpoint that we developed the entropy field decomposition (EFD)^31,32^.

An intuitive way to conceptualize the EFD is by comparison and analogy with Fourier analysis. For example, one version of a ’perfect’ Rossby wave would be a velocity field spatially varying in a perfectly periodic fashion as a function of longitude with an amplitude in the latitudinal direction varying periodically with time. This is an example of a *coherent spatiotemporal structure*. If there were simultaneous waves of different spatial extent, wave frequencies, and amplitude variations, it would be quite difficult to visualize, much less quantify, these different spatiotemporal structures by visual inspection of the data on a weather map. However, a Fourier analysis would be able to do this. The “modes” of velocity would be the 3-dimensional (in space) time courses (i.e., time-dependent volumes) reconstructed from the Fourier components with the largest amplitudes, ranked in decreasing order. There may be many “modes” and determining their relative significance in a standard analysis would be done by sorting the Fourier amplitudes in decreasing order. Determining where the modes cease to be significant is a more complicated question that depends on the noise in both the system and the measurements.

If, in addition to these velocity field spatiotemporal patterns, there were other periodically varying (in both space and time) physical parameters, for example, humidity, whose variations were related to (perhaps in a complicated way) the spatiotemporal variations in the velocity field, then a Fourier analysis could detect the spatial and temporal variations of the moisture field. Determining the relationship to the wind field poses a more complicated problem that requires determining the often non-linear relationship between the different parameter fields.

The EFD can be thought of in direct analogy with a Fourier analysis. A Fourier analysis consists of expressing a signal in terms of its contribution from the Fourier functions, which are sinusoids. This is an expression of the implicit assumption that the signal is modeled as periodic. The Fourier space-time modes are easily calculated because the contributions of the different so-called Fourier “basis” functions (plane waves of a particular frequency) to the signal are added independently (the functions are perpendicular, or “orthogonal”, to one another).

However, in highly non-linear, non-periodic, and non-Gaussian physical systems like the weather, there is no obvious set of basis functions to use for computing the independently contributing modes. The major significance of the EFD method is that it performs the same decomposition of the data into space-time modes that show the dominant spatiotemporal patterns ranked in order of the amplitude of modes coefficient.

In our view, these problems fall within the domain of probability theory, from which EFD was derived from first principles. Indeed, as we show in *Appendix A* the general EFD formulation reduces to the probabilistic formulation of the Fourier solution when less prior information is used.

But what makes the EFD such a methodology is that it can determine the unique set of basis functions within each individual dataset directly from the structure of the data itself, and works for complex non-Gaussian, non-linear and non-periodic spatiotemporal patterns not known *apriori*. This can be accomplished formally via the theory of entropy spectrum pathways (ESP) that determines the unique set of orthogonal space-time basis functions directly from the space-time correlation structure of the data. The end result is therefore a set of space-time modes (“eigenmodes”) ranked in order of amplitude of the mode coefficients (“eigenvalues”). The procedure, however, is then completely analogous to a Fourier decomposition into different space-time modes.

The EFD methods can be extended to multiple modalities by incorporating coupling between different parameters, which we call *Joint Estimation with Entropy Regularization* (JESTER)^45^. This allows us to perform this analysis simultaneously for datasets with multiple data fields, such as wind and humidity, and determine the ranked modes for spatiotemporal variations of the mutually interacting fields. These are the space-time modes that are demonstrated in this paper. A brief introduction to the logical extension of EFD to multiple modalities is provided in *Appendix A*.

Because the EFD analysis is formulated within the logical structure of Bayesian field theory, the resulting modes provide *quantitative* information including the detailed spatial-temporal patterns and the relative contribution of the different modes. This information obviously cannot be discerned by visual inspection of weather maps.

We note that while methods that decompose data into components or modes are often referred to as “data reduction” techniques, we find that term somewhat misleading as the data are not reduced, but rather quantitatively refined into probability subspaces. But in some sense this is just semantics.

## Entropy field decomposition (EFD)

**Theory.** The EFD is a probabilistic method for estimating spatial-temporal modes of complex non-linear systems containing interacting fields (e.g., wind, humidity, temperature, pressure, etc). It is formally based on a field-theoretic mathematical formulation of Bayes’ Theorem that enables the hierarchy of multiple orders of field interactions including coupling between fields^31,32^. Its practical utility is enabled by incorporation of the theory of entropy spectrum pathways (ESP)^46^ which uses the space-time correlations in each individual dataset to automatically select the very limited number of highly relevant field interactions. There are no training datasets or averages across datasets—just the prior information contained within the single dataset of interest. This method has shown utility in meteorology in the application to severe local storms, in particular tornadic supercells^33^. In addition to producing estimated field modes, the EFD facilitates the construction of space-time information trajectories (STIT) which are paths of optimal (in the sense of maximum entropy) information within any estimated EFD mode. Multiple connectivity eigenmodes (CEM) generated from each STIT characterize the primary information pathways in the data. (As noted in the previous section, *Appendix A* provides a brief introduction to the logical development of EFD and STITs.) The computational implementation is detailed in *Appendix C*. In the results that follow, it will be noted that STITs reveal wave-like patterns in the atmosphere. Since the STITs are generated by the coupling of the wind field to other dynamical parameters (e.g., humidity), they will be necessarily related to atmospheric Rossby waves that are traditionally calculated directly from the wind field, often by standard tracking wave paths (e.g.,^47^. STITs patterns are similar to Rossby Waves, because STITs are greatly impacted by the wind fields, while Rossby Waves are closely associated with the large-scale wind fields. In other words, STITs captured the pattern of planetary-scale circulation (STITs are similar to the pattern of Rossby Waves). Given the connection between ARs (EFD modes) and STITs, it reveals a link between ARs and planetary-scale circulation. It is worth noting that STITs are not Rossby Waves, they are optimal space-time path probabilities based on the specific humidity and wind field configurations.

## Case studies

### Three AR cases

While ARs occur worldwide, our particular interest is in those that impact the Western US, as they play a critical role in the precipitation over this region and have great impacts on extreme precipitation, flooding, and landslide. We have therefore chosen two particular damaging storms that hit the Western US: (1) landfalling AR over the U.S. Pacific Northwest on 26th March 2005; (2) landfalling AR over the California coast on 27th January 2021. Both were major landfalling AR events over the West Coast and produced heavy precipitation in the corresponding regions. In addition, as a third case study we consider a historical event that is famous for its hurricane-force wind—the Great Storm of 1987 that occurred on 15th-16th October 1987 over the UK. Indeed, the term “atmospheric river” (which appears to have been used for the first time by Zhou^48^) had not even been coined at that time. Rather, the infamy of this storm was not just the rapidity of the formation of severe weather—it was the most damaging storm in 200 years in UK and caused almost $2b in damage and killed 19 people, but also for the failure of the meteorologists to predict it^49^. The well documented highly localized (in both space and time) nature of the event makes it appealing for a more in-depth test case for our EFD space-time analysis method. It also provides for an interesting comparison with other studies of ARs in this region, e.g.,^50^.

### Data sources

**Figure 1:**
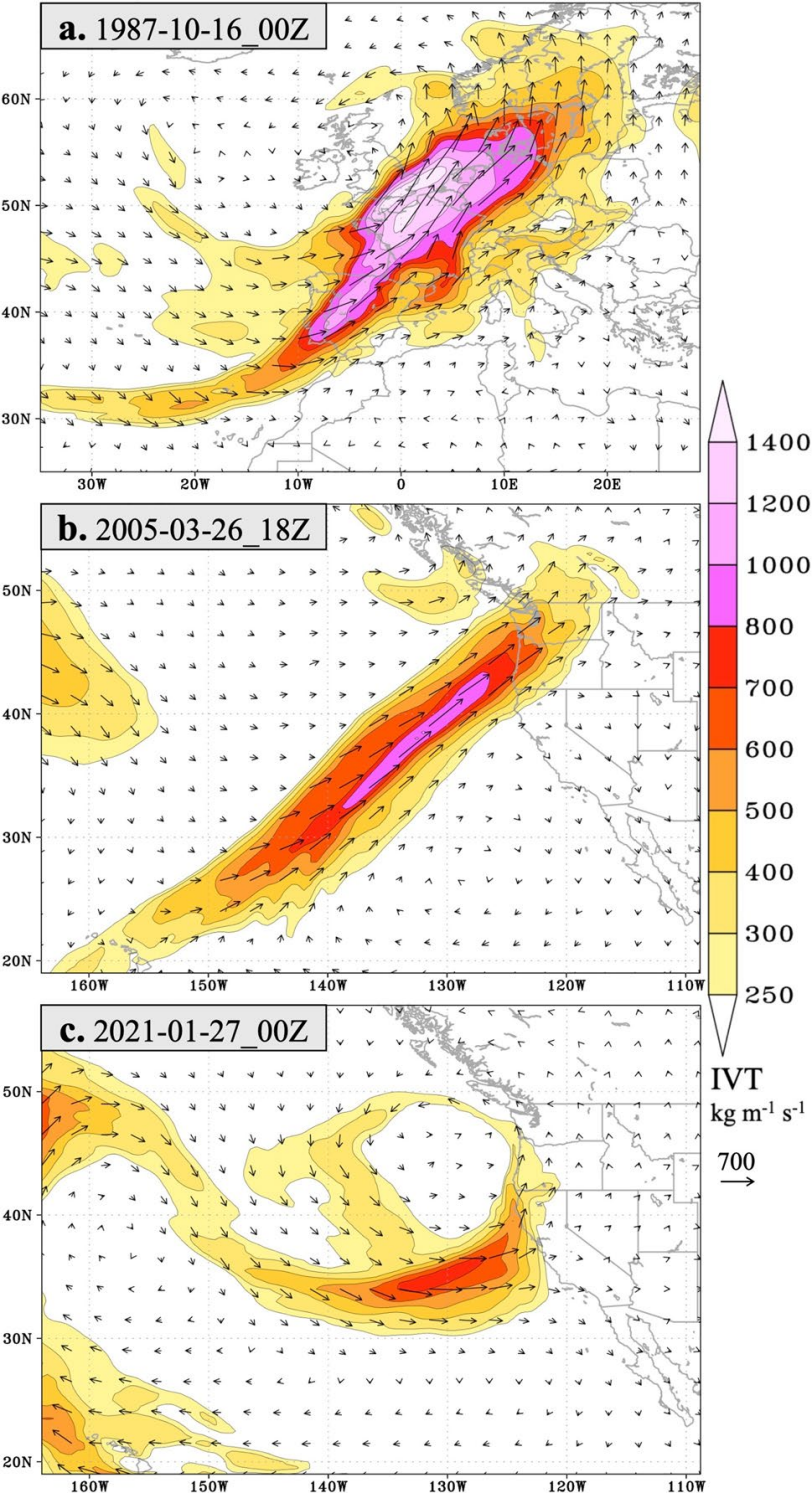
Integrated vapor transpose (IVT) for the (Top) AR of Mar2005; (Middle) AR of Jan2021; (Bottom) Great Storm of 1987 at 18 UTC on Oct 15th based on NCEP CFSR Reanalysis.

In this study, the Climate Forecast System Reanalysis (CFSR,^51^) dataset from the National Centers for Environmental Prediction (NCEP) is used for the analysis of the October 1987 and the March 2005 AR case. Since CFSR spans from January 1979 to December 2010, the NCEP Climate Forecast System Version 2 analysis^52^ is used as the extension of CFSR after January 2011 and here for the January 2021 AR case analysis. The CFSR data was obtain on 6-hourly temporal resolution, 0.5$^\circ$ longitude $\times$ 0.5$^\circ$ latitude horizontal resolution, and 19 pressure levels (from 1000 hPa to 100 hPa with 50-hPa interval). The variables used in this study include specific humidity (Q), zonal and meridional wind components (U &V), and vertical wind velocity. Vertically integrated water vapor transport (IVT) was calculated using Q and U &V from 1000 to 100 hPa following^18^. For each case, an extended period (10 days) of data was utilized to cover the entire life cycle of ARs, starting from 11th October 1987 for the 1987 case, from 21st March 2005 for the 2005 case and from 22nd January 2021 for the 2021 case, respectively. The IVT for the three case studies in this paper are shown in Fig. 1 and clearly depict ARs.

## Results

In what follows below, we will denote the calculated *k*’th EFD mode of the coupled parameters $\varvec{\alpha } = \{ \alpha _{1},\ldots ,\alpha _{m} \}$ as $\psi ^{(k)}_{\varvec{\alpha }}(\varvec{x},t)$. The STIT generated from the modes will be denoted $F^{(k)}_{\varvec{\alpha }}(\varvec{x},t)$. In this paper, the *k*’th mode was generated by *k*’th nearest-neighbor in both space and time. However, the algorithm more generally supports independent variations in coupling $\psi ^{(k,l)}$ where $k=0,\ldots ,k_{m}$ and $l=0,\ldots ,l_{n}$ represent the coupling length for space and time, respectively, so that an $m \times n$ array of space-time modes with different combinations of space and time scales can be constructed. This provides a refined analysis of spatio-temporal phenomena at different scales and will be pursued in the present context in the future. Our primary interest here is investigating the hypothesis that ARs can be revealed and characterized from EFD modes computed from the (3D+t) wind velocity $v(\varvec{x},t)$ coupled with the specific humidity $q(\varvec{x},t)$ fields.

For AR events highly localized in both time and space, such as the Oct 1987 storm the lowest order EFD mode $\psi ^{(0)}_{v,q}(\varvec{x},t)$, (i.e., nearest neighbors in space and time) which emphasizes the smallest spatial and temporal scales, should be most sensitive to ARs. And the corresponding STITs $F^{(0)}_{v,q}(\varvec{x},t)$ computed from this mode will reveal the larger scale dynamical system influencing these rapidly evolving events. However, for ARs that evolve over broader ranges of space and time, such as the Jan 2021 event, higher order modes (e.g. $\psi ^{(2)}_{v,q}(\varvec{x},t)$ and the corresponding STIT $F^{(2)}_{v,q}(\varvec{x},t)$) reveal the storm dynamics more clearly.

### Western US AR cases

The March 2005 AR case (Fig. 1A) had a tropical moisture source over the southwest of the Hawaiian Islands, extended northeastward to the U.S. Pacific Northwest, and made landfall over the Washington and north Oregon coast on 26th March with precipitation over 100 mm in the following 24 h^21^. The January 2021 AR case (Fig. 1B) formed on the south side of a deepening extratropical cyclone over the eastern North Pacific, made landfall over the coastal Northern California on 27th January, and then migrated southeastward along the coast of California. This case produced heavy precipitation over a large area of California with a maximum over the Central California and brought severe floodings, which is one of the Billion-Dollar Weather and Climate Disasters in 2021 according to the report from National Oceanic and Atmospheric Administration^53^. These two cases are typical ARs over the eastern North Pacific and the U.S. West Coast, but with some diversities in their characteristics and the large-scale background.

**Figure 2:**
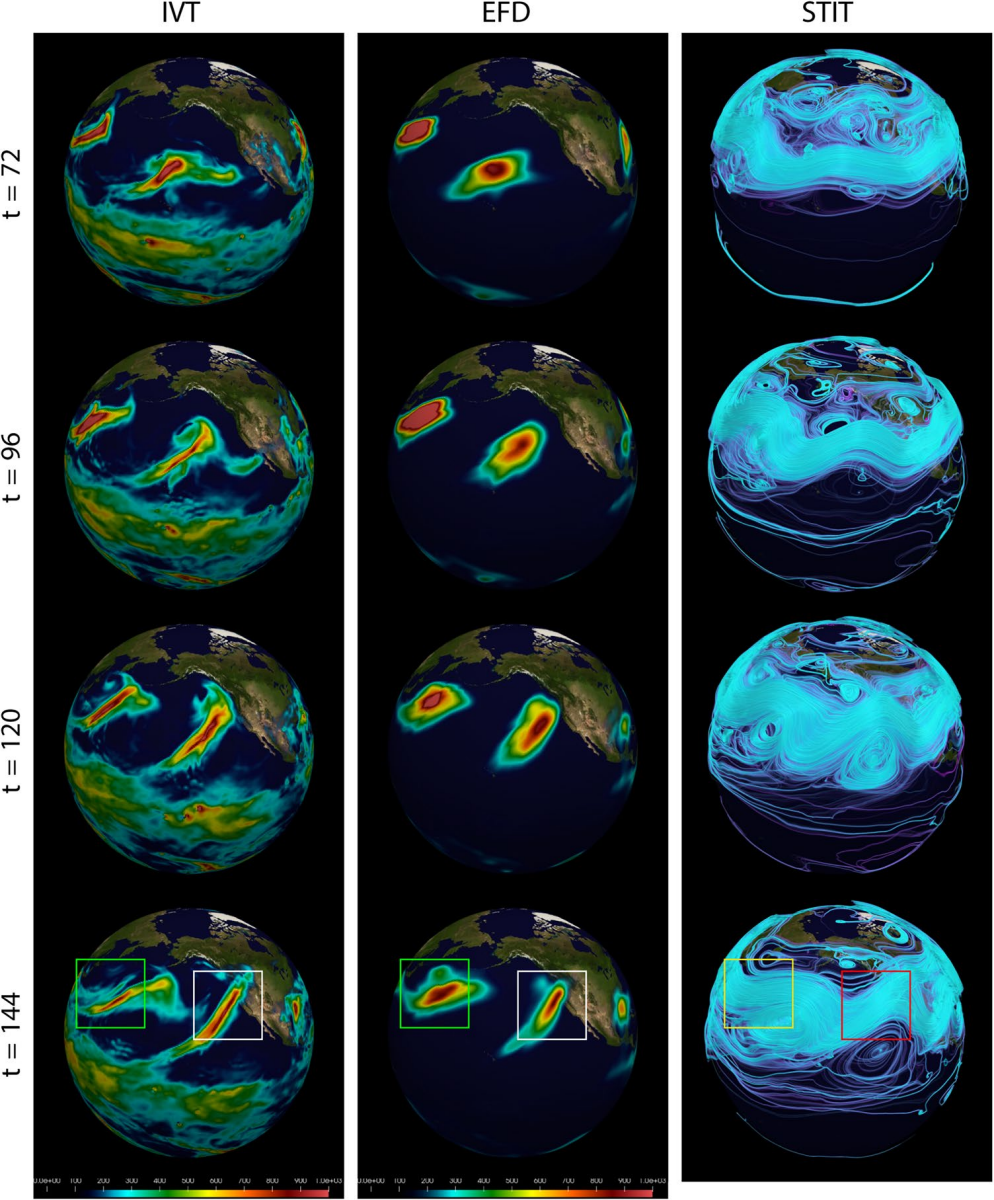
Spatiotemporal evolution of the AR and its relationship to amplification of the STIT waves for the Mar 2005 event computed from all three spatial dimensions and the time dimension (denoted “3D+t”). Time points for Mar2005 shown are $t=\{72,96,120,144\}$. (Left column) IVT, (Middle column) Maximum intensity project of the (3D+t) EFD mode $\psi ^{(0)}_{v,q}(\varvec{x},t)$ computed using only the (3D+t) wind field coupled with the (3D+t) specific humidity field, (Right column) STITs $F^{(k)}_{\varvec{\alpha }}(\varvec{x},t)$ generated from the EFD mode $\psi ^{(0)}$, along with a $\psi ^{(0)}$. The AR that impacted CA is shown in the white boxes (IVT and EFD), and appears to be associated with the STIT wave trough in the red box. A second AR is labeled using the green boxes (IVT and EFD) and the yellow box (STIT). STIT coloring is arbitrary and optimized for visualization. EFD and STIT scale is unitless. Time is hours since 2005-03-21 00Z.

**Figure 3:**
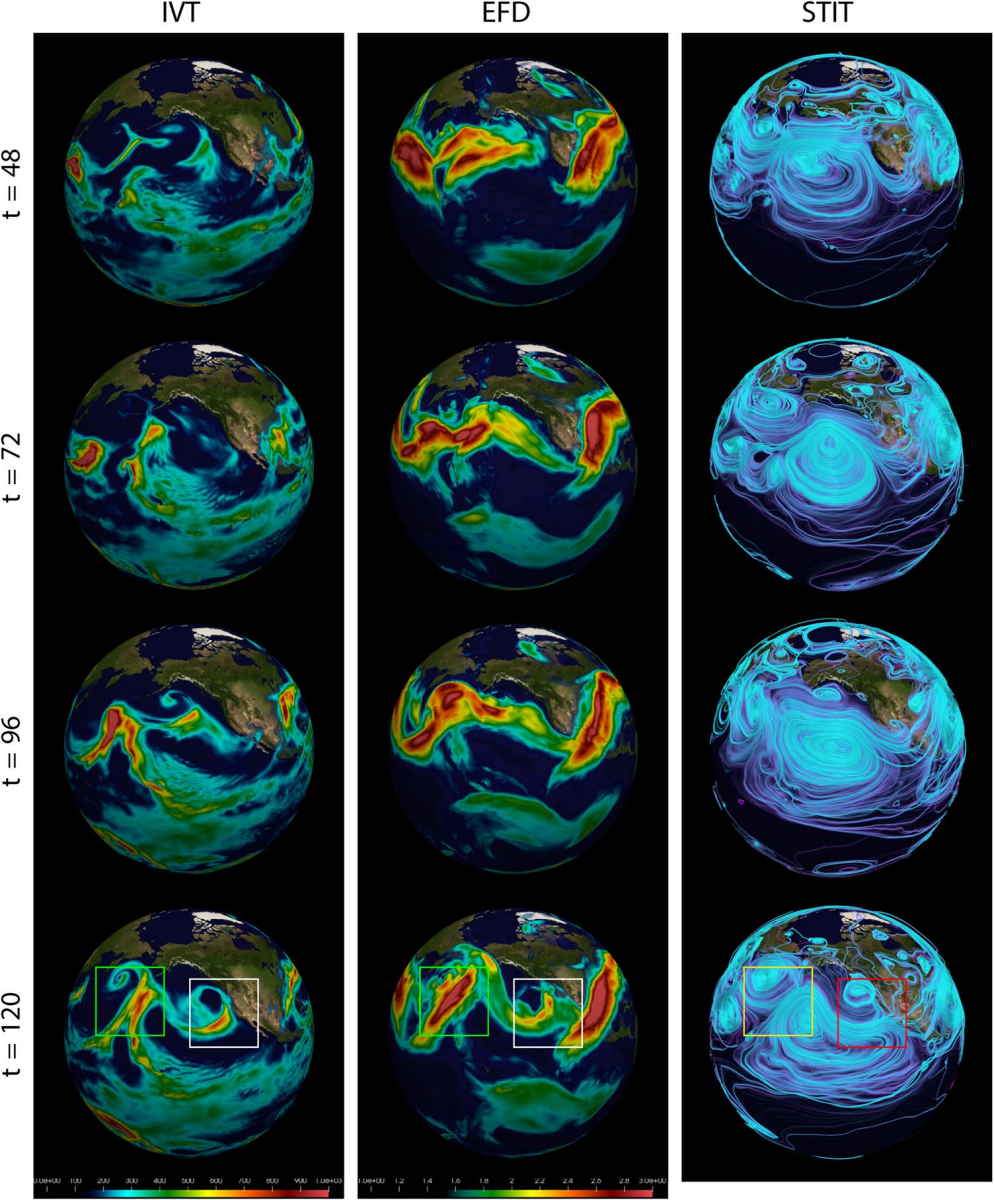
Time evolution of the AR and its relationship to amplification of the STIT waves for the Jan2021 events. Time points for Jan2021 are $t=\{48,72,96,120\}$. (Left column) IVT, (Middle column) Maximum intensity project of the (3D+t) EFD mode $\psi ^{(2)}_{v,q}(\varvec{x},t)$ computed using only the (3D+t) wind field coupled with the (3D+t) specific humidity field, (Right column) STITs $F^{(k)}_{\varvec{\alpha }}(\varvec{x},t)$ generated from the EFD mode $\psi ^{(2)}$, along with a $\psi ^{(2)}$. The AR that impacted CA is shown in the white boxes (IVT and EFD), and is appears to be associated with the STIT wave exit region in the red box (STIT). The depth of the STIT wave, and the corresponding steepness of the exit region, are correlated with the strength of the AR as estimated by EFD. Indeed, the AR that hit CA was significantly less powerful that the Western AR over the North Pacific (labeled with green and yellow boxes). STIT coloring is arbitrary and optimized for visualization. Time is hours since 2021-01-22 00Z.

The EFD modes were calculated over all time frames ($t=0,6,12,\ldots , 234$ hours in steps of 6 hours) spanned from 00 UTZ 21st to 18 UTZ 30th March 2005 for the Mar 2005 event and from 00 UTZ 22nd to 18 UTZ 31st January 2021 for the Jan 2021 event. The results for the Mar 2005 event are shown in Fig. 2 and for Jan 2021 in Fig. 3. Four key time points in the evolution of the ARs is shown: (1) generation, (2) development, (3) enhancement, and (4) landfall. These correspond to times $t=72, 96, 120, 144$ for the Mar 2005 event and $t=48, 72, 96, 120$ for the Jan 2021 event. ARs are often identified and characterized using IVT, which is shown in left column of Figs. 3 and 2. In the second column of these figures is shown the EFD modes and in the right column display the STITs. For the March 2005 event the first EFD mode $\psi ^{(0)}_{v,q}(\varvec{x},t)$ and the corresponding STITs $F^{(0)}_{v,q}(\varvec{x},t)$ computed for this mode are displayed in Fig. 2. For the Jan 2021 event the third EFD mode $\psi ^{(2)}_{v,q}(\varvec{x},t)$ and the corresponding STITs $F^{(2)}_{v,q}(\varvec{x},t)$ computed for this mode are displayed in Fig. 3. This higher mode corresponds to longer range spatial and temporal coupling, and show greater sensitivity to the AR in the Jan 2021 event because of its more extended spatial structure and slower time evolution. As a reminder, though, all modes are calculate for all events, and from a practical perspective are all useful in more fully understanding the dynamics of AR events.

The wind and specific humidity at the 850 hPa pressure level from these events are shown at the same time points in Figs. 14 and  15 in *Appendix B*. Note that the IVT is an integrated 2D spatial field whereas the EFD is a full (3D+t) spatiotemporal field, so provides information as function of altitude. Full volume rendering of this field can be visually confusing, however, so for display purposes, we display the maximum intensity projection (MIP) over all vertical coordinates in Figs. 2 and 3 as the EFD modes to compare with the traditional IVT fields.

In the 2005 case, a well-defined AR appeared in the IVT field (left column of Fig. 2) at $t=72$ over the middle North Pacific, where a strong low-level jet captured the water vapor from the southwest of the Hawaiian Island (Fig. 14). Then the AR developed and propagated northeastwards in the next two days (t = 96 and t = 120), until it made landfall over the U.S. Pacific Northwest at t = 144. The EFD mode (middle column in Fig. 2) successfully revealed the AR through its life cycle, from the genesis stage to landfalling. The EFD modes have been scaled to emphasize their subtle and complex structure. The most intense regions of EFD modes correspond directly to the ARs detected by IVT.

It is noteworthy that the EFD mode is calculated using the raw wind and humidity fields directly. It estimates the spatial-temporal modes of the complex non-linear system with the interacting wind and humidity fields, rather than the water vapor transport field (IVT)), which is only a measurement of horizontal water vapor transport. However, the EFD mode still can capture the AR patterns, which are very similar to the AR objects in the IVT fields (right column in Fig. 2). It implies that the AR is not only a strong horizontal water vapor transport corridor, but also a region with intense interaction between the wind and water vapor.

Different from the 2005 case, the 2021 case formed at the relatively higher latitudes in the middle North Pacific, moved eastward, and made landfall at the California coast (Figs. 3 and 15). The EFD modes also revealed the AR patterns, which are similar to the AR objects in the IVT field. These results indicate that the EFD mode can provide an alternative metric to identify ARs based on the interaction between wind and water vapor. In addition to the EFD mode, the right columns of Figs. 2 and 3 show the STITs. STITs are high probability pathways through the joint wind-specific humidity EFD mode and is highly related to the upper-level wind field, which could be used to identify Rossby waves. Thus, STITs exhibit a pattern consistent with Rossby waves. Both AR cases are located at the southeast of the wave trough, where the strong low-level southwest wind advects the water vapor from the subtropical/tropical region to the middle latitudes (Figs. 14 and 15). It indicated that the formation of the two ARs are related to the amplified STIT wave trough.

In the case of the Jan2021 event, a much stronger AR is seen to the west over the North Pacific Ocean, which can be seen in both IVT field and EFD mode (t=120 in Fig. 3). Its greater strength correlates with the significantly larger depth of the trough of the associated amplified STIT wave relative to the AR that made landfall in California.

In addition to the main AR cases, EFD also detects a second AR to the East for both cases. While our initial interest was in assessing our ability to detect the major AR events that impacted the US West Coast, the detection of the second AR is important in supporting our hypothesis, derived from the STIT $F^{(0)}_{v,q}(\varvec{x},t)$ in right hand panels, that these ARs form as a result of convergence of water vapor transport along the southeast region of the trough in what appear to be amplified STIT waves. Both second ARs appear in these regions of the STIT waves. The apparent amplification of the STIT waves and their relationship to the AR development is evident in the several time frames of the EFD mode $\psi ^{(0)}_{v,q}(\varvec{x},t)$ and STIT $F^{(0)}_{v,q}(\varvec{x},t)$ for both the Mar 2005 event shown in Fig. 2 and for the Oct 1987 even shown in Fig. 5, and in $\psi ^{(2)}_{v,q}(\varvec{x},t)$ and STIT $F^{(2)}_{v,q}(\varvec{x},t)$ for the Jan 2021 event shown in Fig. 3. It is reasonable since a southwesterly low-level jet stream ahead of the cold front of an extratropical cyclone, which is usually located at the downstream of Rossby Wave trough, can provide a favorable condition for the formation and development of ARs^24^.

**Figure 4:**
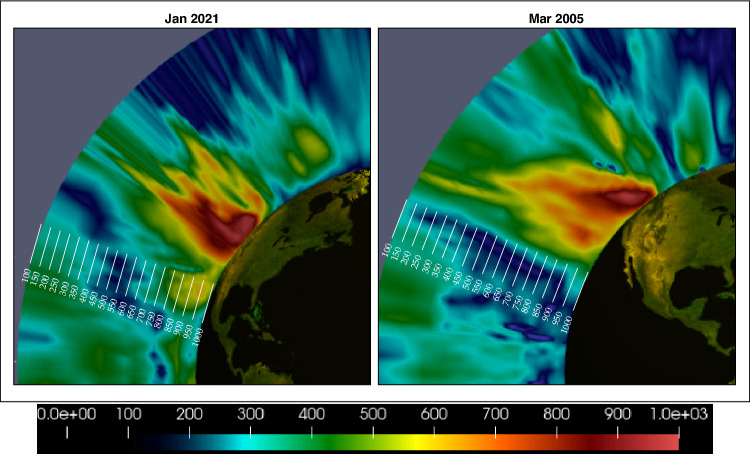
Vertical structure of the EFD mode $\psi ^{(0)}_{v,q}(\varvec{x},t=144)$ at $122^\circ$ W over West Coast of the Continental US for the Jan 2021 event (Left) and the Mar 2005 event (Right) emphasized the subtle volumetric variations provided by EFD. The scale of the vertical measurements has been exaggerated by a factor of 10 for visibility. Units for pressure are hPa. EFD scale is unitless.

**Figure 5:**
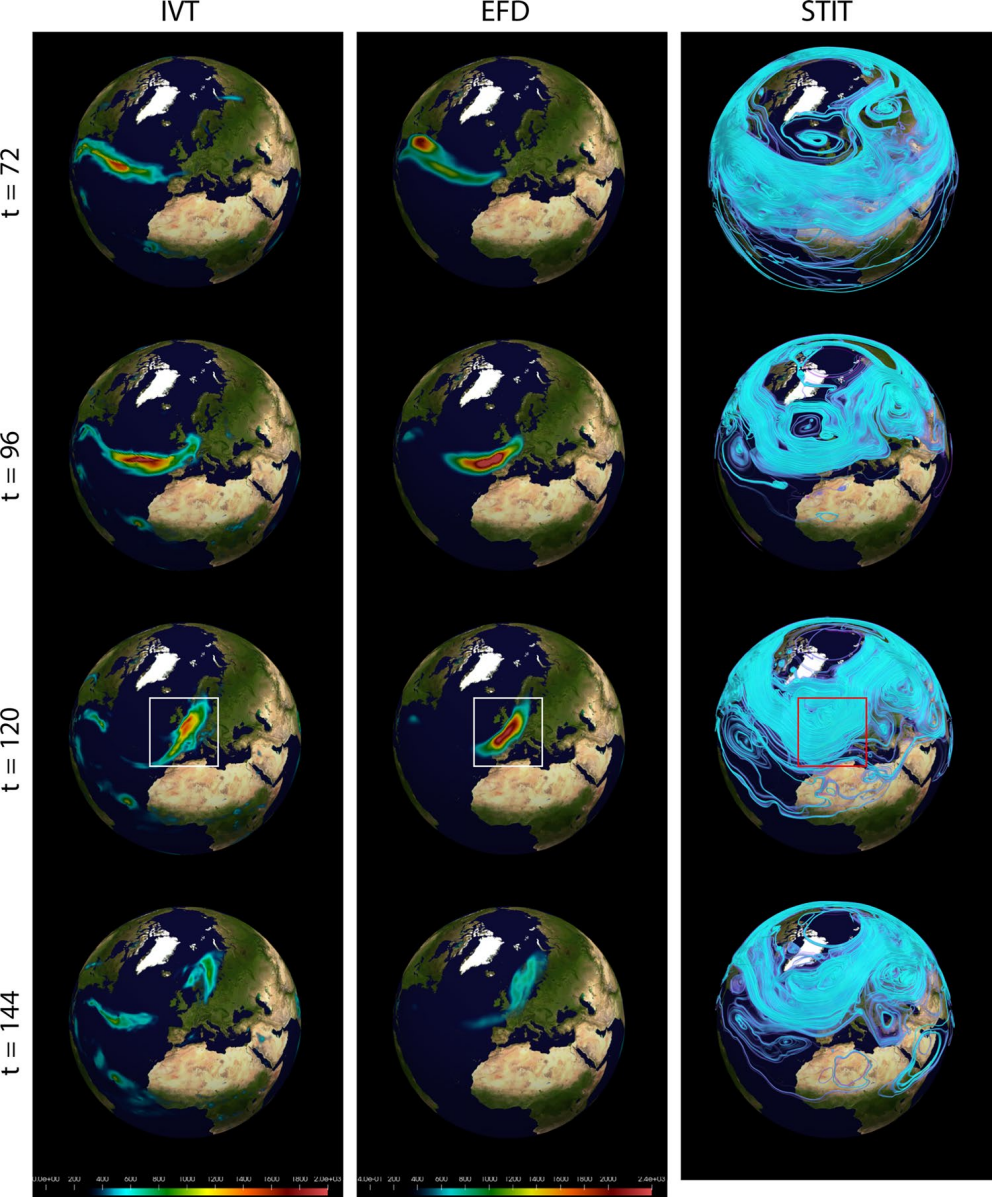
Time evolution of the AR and relationship to amplification of the STIT waves for the Oct 1987 event. Time points for Oct 1987 shown are $t=\{72,96,120,144\}$. (Left column) IVT, (Middle column) Maximum intensity project of the (3D+t) EFD mode $\psi ^{(0)}_{v,q}(\varvec{x},t)$ computed using only the (3D+t) wind field coupled with the (3D+t) specific humidity field, (Right column) STITs $F^{(k)}_{\varvec{\alpha }}(\varvec{x},t)$ generated from the EFD mode $\psi ^{(0)}$ automatically localizes the AR. The AR is shown in the white box (IVT and EFD) at time of landfall in the UK ($t=120$) and is coincident with the ejection region of an amplified STIT wave (red box). STIT coloring is arbitrary and optimized for visualization. Time is hours since 1987-10-11 00Z.

The volumetric nature of the EFD estimates is emphasized in Fig. 4 where slices through $\psi ^{(0)}_{v,q}(\varvec{x},t=144)$ approximately perpendicular to the primary orientation of the two AR events. The EFD mode exhibits a maximum from the surface to $\approx 800 hPa$ for both cases, which indicates that the wind-water vapor interaction is mainly concentrated at the lower levels. This pattern is consistent with the vertical structure of ARs in previous studies based on IVT (e.g.,^21^), showing that the water vapor transport occurs mainly in the lower troposphere.

The wind and specific humidity fields at the same time points discussed in the EFD results in "Results" section are shown in (Figs. 14 and 15) for the Mar 2005 and Jan 2021 events, respectively.

### The great storm of 1987

Although the Great Storm of 1987 was intensively discussed for its hurricane-force wind, it also has an associated exceptionally strong AR with a maximum IVT over 1250 kg m^-1^ s^-1^ around the English Channel (Fig. 1C). The exact same automated EFD analysis used for the Western US cases was thus applied to the (3D+t) data from this event.

As before, the EFD modes were calculated over all time frames ($t=0,6,12,\ldots , 234$ hours in steps of 6 hours) spanned from 00 UTZ 11st to 18 UTZ 20th Oct 1987 for the Oct 1987 event. Fig. 5 shows the development of this AR across the North Atlantic ($t=72, 96, 120, 144$). The IVT is shown in the left column of Fig. 5. In the second column is shown EFD mode $\psi ^{(0)}_{v,q}(\varvec{x},t)$ (middle panel) again computed from the (3D+t) wind velocity $v(\varvec{x},t)$ and specific humidity $q(\varvec{x},t)$ fields. The left column displays the STITs $F^{(0)}_{v,q}(\varvec{x},t)$ computed for this mode. The wind and specific humidity at the 850 hPa pressure level from these events are shown at the same time points in Fig. 16 in *Appendix B*. Again, although the EFD is a full (3D+t) spatio-temporal field, we display the maximum intensity projection (MIP) over all vertical coordinates in Fig. 5 as the EFD modes to compare with the traditional 2D+t IVT fields.

The AR associated with the Great Storm of 1987 is seen in the IVT field (left column in Fig. 5) to develop in $t=72,96$ in the North Atlantic , impact the UK at $t=120$ , and then proceed up to Norway coast at $t=144$. The full (3D+t) EFD result in Fig. 5 (middle column) derived again directly from the coupled wind and humidity fields, very clearly detects the dynamics of the full (3D+t) event, which is located at the south-southeast of the Rossby wave trough. This is confirmed by the STITs derived from these EFD modes shown Fig. 5 (right column). As in the Western US cases, the maximum intensification occurs at the leading edge of the Rossby wave trough ($t=120$) in concert with amplification of the Rossby wave. The deleterious impact was a result of this intensification being coincident in space and time with landfall over the UK. The wind and specific humidity fields at the same time points from which the EFD results are derived are shown in (Fig. 16).

**Figure 6:**
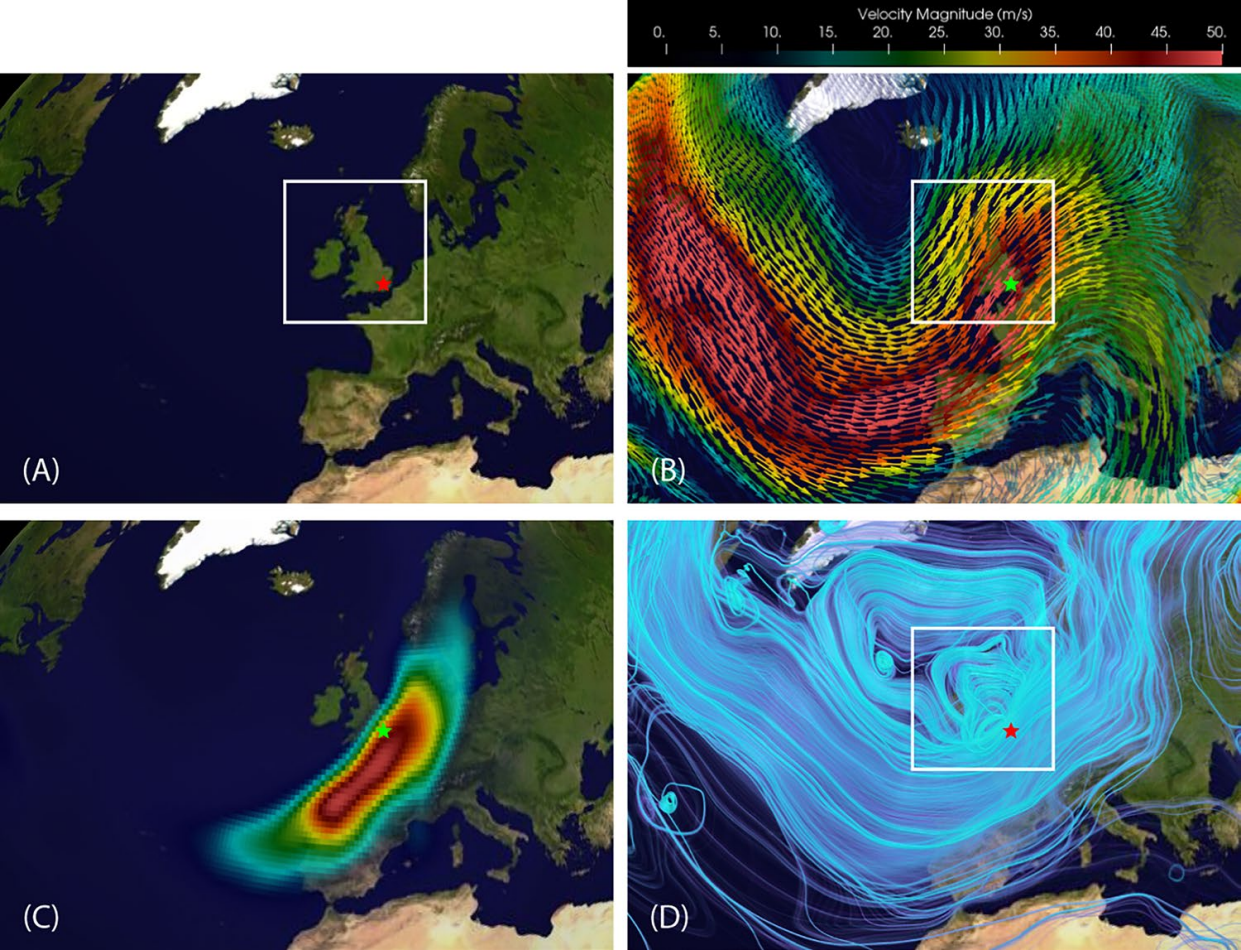
Close-up view of EFD computations for the Great Storm of Oct 1987 at a single time point ($t=120$). (**A**) Map of ; (**B**) Velocity field; (**C**) MIP of EFD mode $\psi ^{(0)}_{v,q}(\varvec{x},t)$ calculated from the (3D+t) wind field coupled with the (3D+t) specific humidity field; (D) STITs $F^{(0)}_{\varvec{\alpha }}(\varvec{x},t)$ generated from the EFD mode $\psi ^{(0)}$, revealing dense trajectories indicative of strong flow, as well as a strong vortex extending all the way to Greenland, and a smaller more intense vortex directly over Ireland/UK with a strong jet on its Eastern edge directly over the coastal region from Plymouth to London. The white box highlights the impacted regions of Ireland and the UK. The star marks the location of the town of Sevenoaks, UK ($51.2724^\circ$ N, $0.1909^\circ$ E) which sits directly in the region of highest wind velocity.

The well documented highly localized (in both space and time) nature of this event makes it appealing for a more in-depth analysis. A close-up view of the EFD results is shown in Fig. 6 and summarizes the capabilities of EFD to reveal and characterize the complex nature of AR events. In Fig. 6A is shown a map of the impacted region. In Fig. 6Bis shown the velocity field at 850*hPa*. The EFD results are shown in the bottom panels. In Fig. 6C is shown the same EFD mode $\psi ^{(0)}_{v,q}(\varvec{x},t)$ as in Fig. 5 but with a different colormap to highlight mode variations within the event localization. The corresponding STIT $F^{(0)}_{v,q}(\varvec{x},t)$ computed for this mode is shown in Fig. 6D.

**Figure 7:**
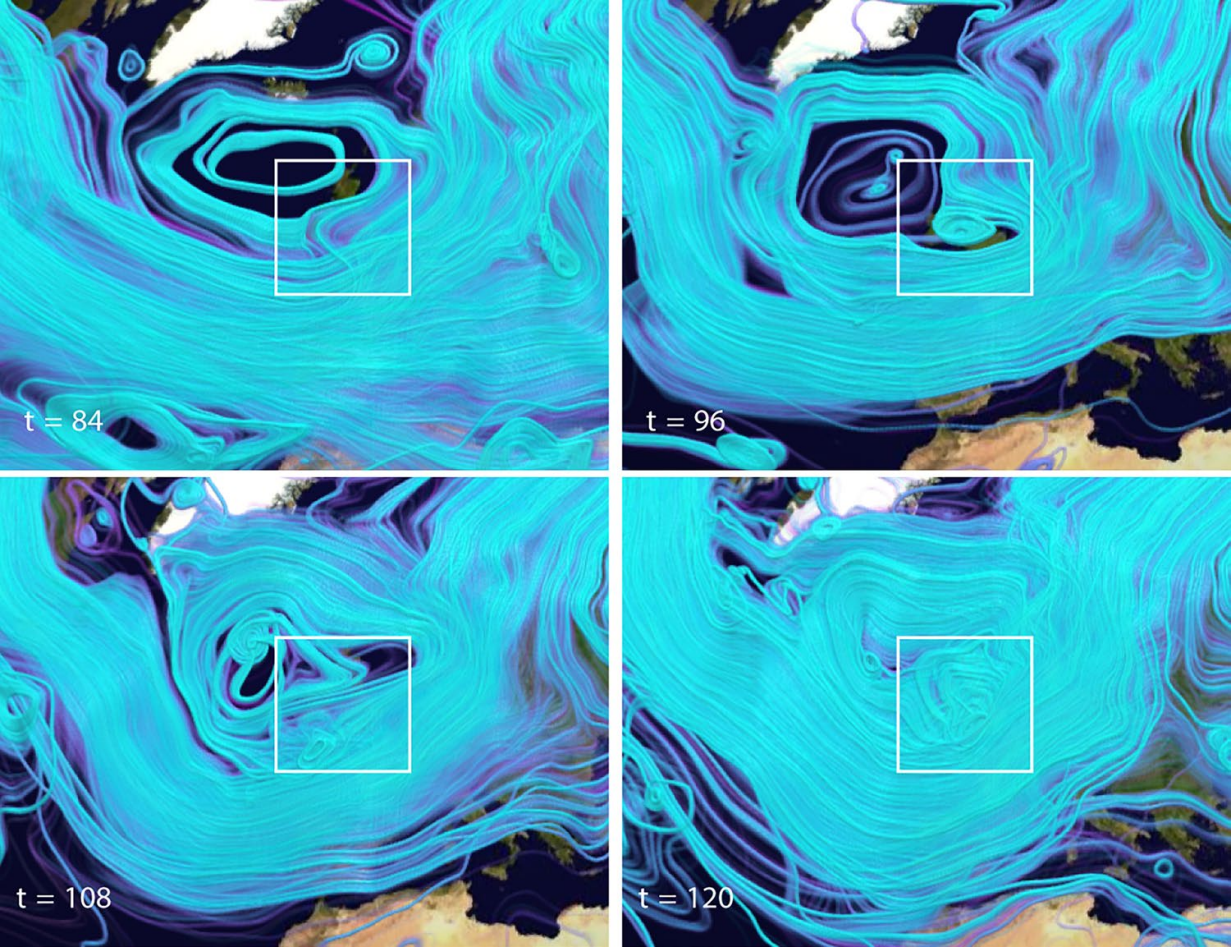
Close-up STITs $F^{(0)}_{\varvec{\alpha }}(\varvec{x},t)$ at four time points leading up to the image in Fig. 6D when the storm was near its maximum intensity at landfall in the UK. These have been rendered with a slightly different opacity to highlight regions of dense trajectories. The rapid development of a strong vortex over the UK is evident. The white box highlights the impacted regions of Ireland and the UK.

The STITs reveal dense trajectories indicative of strong flow, as well as a strong vortex extending all the way to Greenland, and a smaller more intense vortex directly over Ireland/UK with a strong jet on its Eastern edge directly over the coastal region from Plymouth to London. The box highlights the impacted regions of Ireland and the UK. The star marks the location of the town of Sevenoaks, UK which sits directly in the region of highest wind velocity and became infamous for the toppling of all seven oaks that gave the town its name. The development of the STITs leading up to this time is shown in Fig. 7 and show a rapid development of the dense vortical trajectories particularly on the trough exit region coincident with the AR.

### Some remarks on the STITs

**Figure 8:**
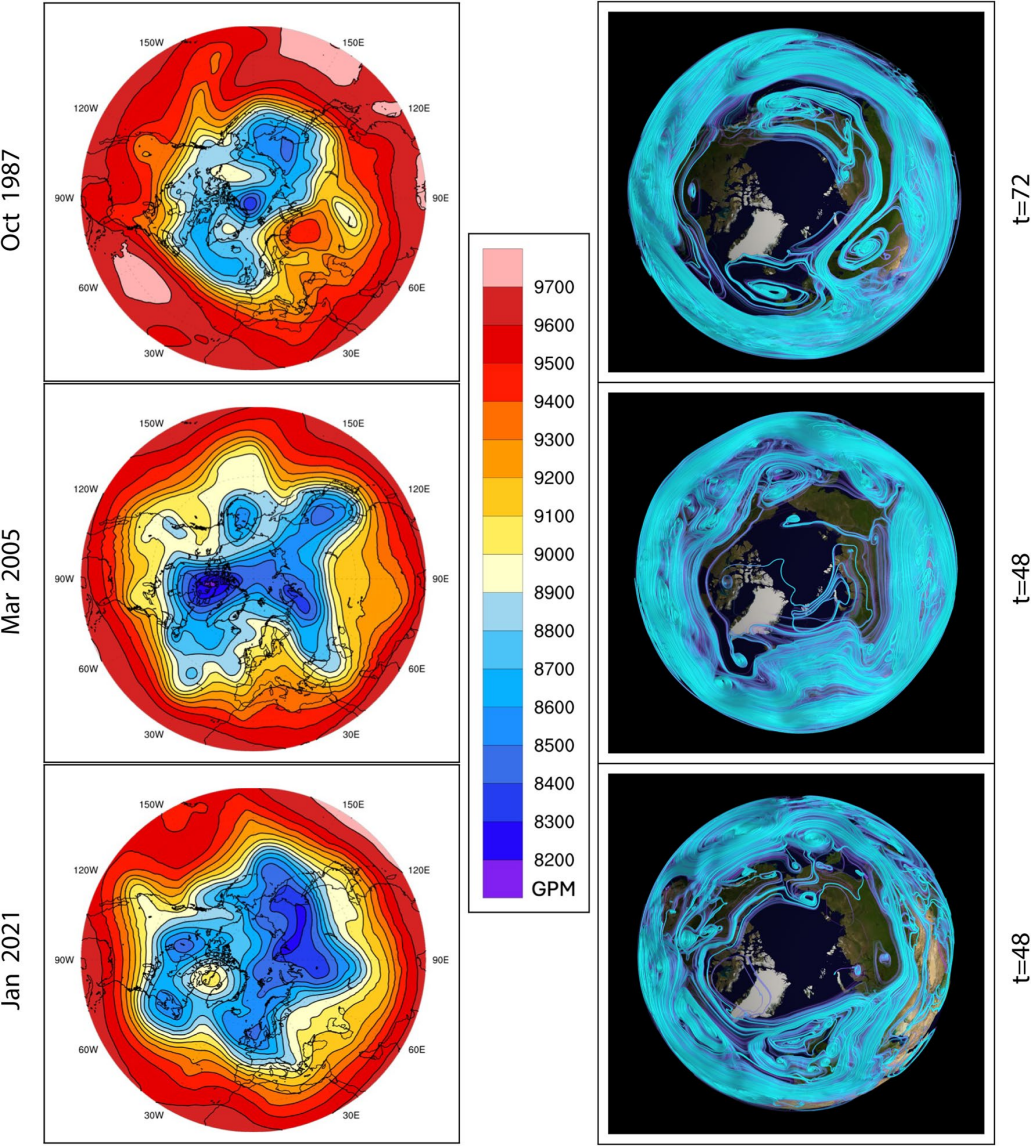
Comparison of the STITs and the geopotential height at 300*hPa* for a single time point for the three storms. The view is directly down on the North Pole ($-z$). EFD and STIT scale is unitless.

STITs in this study are the optimal space-time path probabilities based on the specific humidity and wind fields configurations. They are greatly impacted by the wind fields, thus their patterns are similar to the patterns of Rossby waves, which are closely associated with the large-scale wind fields. For the data analyzed in this paper, the observed ARs are located along the outflow pathway of the STIT trough, which is consistent with the expected dynamics where the airflow can extract the water vapor from the lower latitudes and transport it to the middle and high latitudes along that outflow pathway. Those outflow pathways are generally very close to the southeast side of the Rossby wave trough, which is closely related to the dynamical factors contributing to ARs, such as Rossby wave breaking and extratropical cyclones^22,23^. It indicates that the results from STITs are consistent with the large-scale dynamical processes associated with ARs. In addition, STITs also take the specific humidity field into account. Therefore, STITs reveal not only the linkage between ARs and large-scale circulation, such as Rossby waves, but also some small-scale factors (regional moisture availability) contributing to the development and propagation of ARs. To emphasize this point, a comparison of the STITs with the geopotential height from a view looking directly down on the North Pole ($-z$) is shown in Fig. 8. It shows that the STITs have similar large-scale patterns with the geopotential height at 300 hPa; meanwhile, they have some regional differences from the geopotential height due to the impacts of specific humidity.

**Figure 9:**
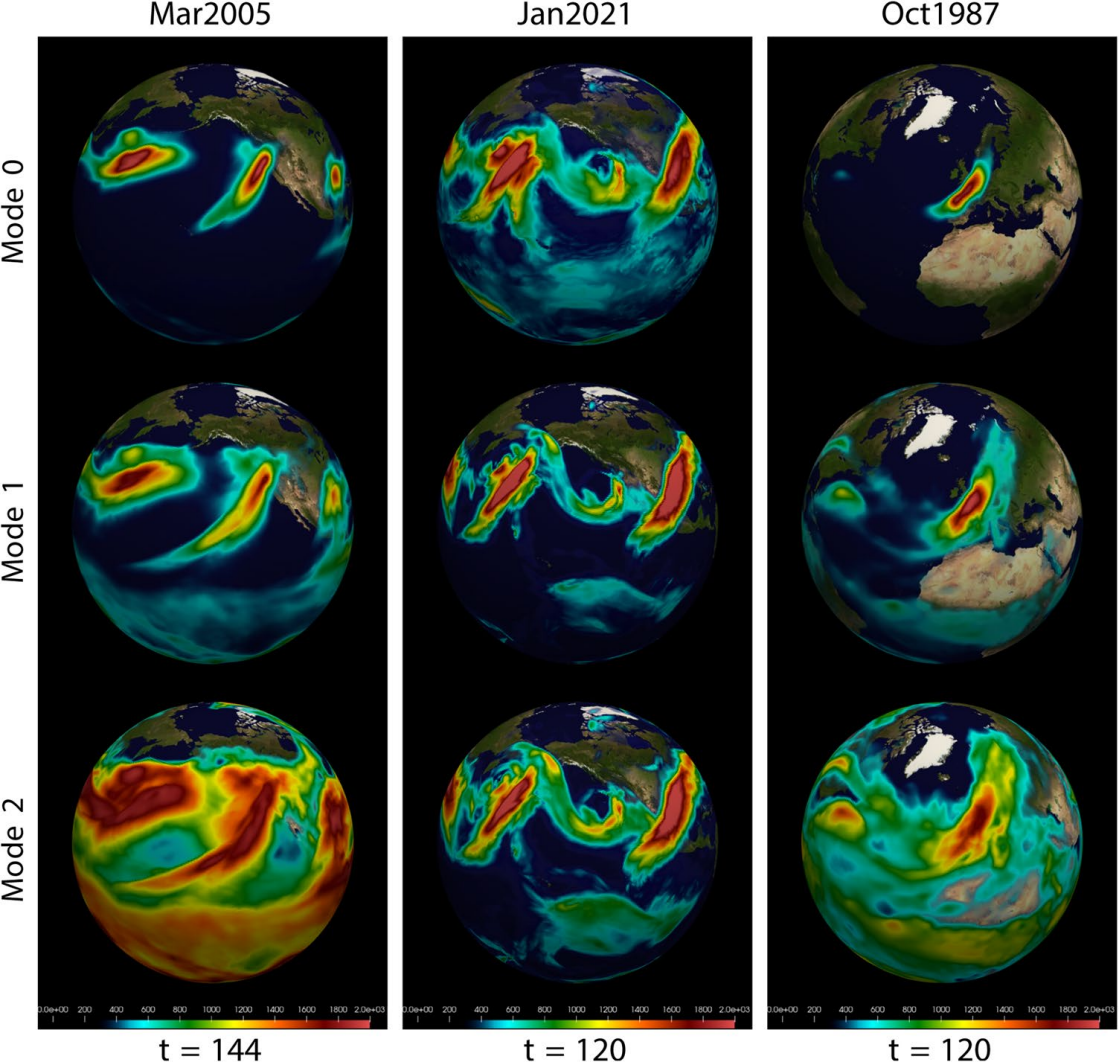
The first three wind-specific humidity EFD modes for a single time point in the three storms, demonstrating that high order modes are sensitive to larger spatial and temporal scales.

## Discussion and conclusion

The rapidly evolving changes to the Earth’s weather accelerated by climate change has put increasing pressure on the meteorological community to develop more accurate methods for characterizing and predicting severe weather events. The continuing rapid improvement in weather measurement technologies provide increasingly complex data which hold the promise of more informative forecasts, but require increasingly sophisticated computational methods to utilize this information. In this paper we have presented two such probabilistic methods, the entropy field decomposition (EFD) and space-time information trajectories (STITs) that provide information not currently available in existing method, and so have the potential to augment the significant amount of ongoing work on global climate meteorology.

We chose to focus the demonstration of our methods on the very specific phenomena of atmospheric rivers (ARs) which are an excellent test case, since they are highly localized in both space and time and yet are formed as a results of the non-linear interaction of a multitude of phenomena at a wide range of spatial and temporal scales. The significant social and economic impact of ARs make their dynamical mechanism and operational prediction of critical importance^10,15^. This in turn necessitates gaining a better understanding of the life cycle—the formation, maintenance, and dissipation—of these complex, multi-scale dynamical events. Major ongoing research efforts are underway to investigate these aspects of ARs (for overviews see e.g.,^10,15,25–28^) and a variety of promising methods have been proposed for their detection and prediction (e.g.,^29,30,54,55^), focusing primarily on spatially localized analysis near the region of AR formation.

In this paper, we introduced a new data analysis framework for investigating the AR’s formation and development, as well as the impacts of large-scale circulation on ARs, that has the potential to add new information on the dynamics of ARs to supplements the major ongoing efforts of research in this area. In particular, we demonstrated the ability to reproduce the life cycle of ARs, from genesis to intensification and then to landfalling, using the Entropy Field Decomposition (EFD) method, and revealed the linkage between large-scale circulation and the propagation of ARs with the Space-Time Information Trajectories (STITs) method. Different from the widely used IVT field, the EFD and STITs methods take into account the multiscale and multivariate nature of the atmospheric fields that might contribute to ARs. An important feature of the general EFD method is that it provides a principled probabilistic formulation of non-linear interacting fields that exist in a wide range of meteorological applications. And given the complexity and uniqueness of individual events, it is a strength of EFD over existing methods that it uses prior information from within *individual datasets* to determine the most probable modes and large scale connectivity, rather than requiring training data from other events, which can bias results in favor of average properties, missing important and unique subtleties evident in any individual event. This will become increasingly important as observation methods (radar, satellite, etc) provide ever more detailed information of system dynamics.

This notion is born out by the results of this study, where we have analyzed three very different major AR events that impacted the Western US, Mar 2005 and Jan 2021, and the significant historical event of The Great Storm of 1987 that impacted the UK. The results are consistent across events and demonstrate that EFD is able to automatically detect and characterize the (3D+t) dynamics of the ARs directly from an analysis of coupled wind-specific humidity fields. In addition, all events reveal a common large scale dynamical feature of formation along the southeast of the Rossby wave trough, as revealed by the STITs. The STITs formalize the “filamentary structure” highlighted in the earliest AR work of^13^ and the trajectory analysis^56^. While not the focus of this paper, this method would provide a method for further investigation of the link between ARs and Rossby waves breaking^22,57^.

The goal of this current paper is to demonstrate the applicability of the EFD/STIT method to global severe weather events. The objective was to focus on a basic analysis in which a single EFD mode was calculated from just two interacting fields, wind and humidity, from which the STIT was then calculated. However, the EFD is a very general procedure in which multiple modes of multiple interacting fields (e.g., wind, temperature, humidity, vorticity, etc) can be computed. These modes are constructed from different spatial and temporal correlation scales and thus can uncover coherences particular to different spatial and temporal scales. This is illustrated in Fig. 9 where three modes are shown for a single time point for each of the severe storms studied above. The construction of the numerical implementation of the EFD/STIT algorithms provide for simple user inputs to specify the number of modes and the spatial and temporal scaling. Of course, increasing the number of modes and extending the analysis across multiple storms will necessarily increase the computational burden, which at some stage requires the memory and speed of supercomputing systems. The multi-threaded nature of the software easily facilitates this, however, and we have implemented this algorithm on two national supercomputer resources. In summary, these extensions should prove useful in the investigation of different storms or different phases of the same storm in both real observations of model simulations and ensembles of models, and will be the focus of future work.

This works suggests that EFD provides a useful new paradigm for detection and quantification of weather patters across all scales of resulting from multiple interacting meteorological fields, and a better understanding of the physics of interacting meteorological fields. For the particular case of ARs investigated in this study demonstrates that an AR is not only a strong horizontal water vapor transport corridor, but also a region of intense dynamical interactions of the important meteorological fields ( i.e., wind and humidity), which can be revealed and quantified with EFD and STITS. Future work points to an investigation the ability of EFD and STIT to characterize the influence of other large-scale phenomena (e.g. MJO) and local phenomena (e.g. orography), and if these techniques can provide a new method for the prediction of ARs and other severe weather phenomena.

## Figures and Tables

**Figure 10:**
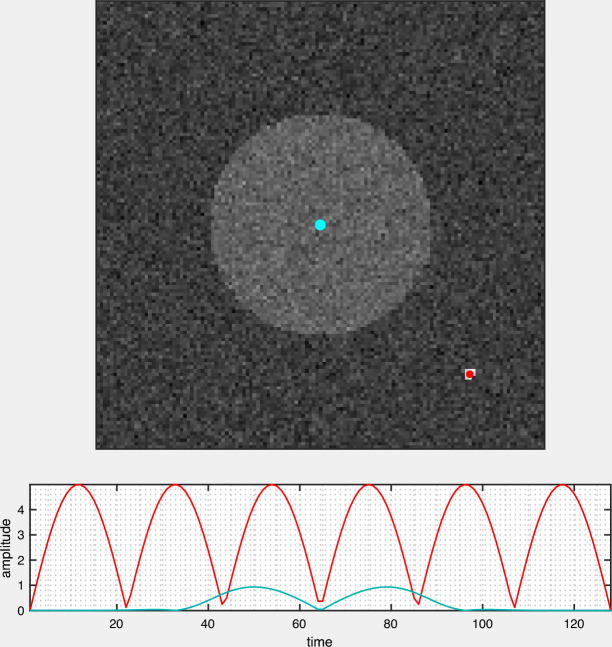
The significance of a detected spatiotemporal pattern. Idealized numerical simulation in which there are two areas of activity in Gaussian random noise: a point oscillating with very high signal-to-noise (SNR) (centered on the red dot) but also a larger circular region with very low SNR (centered at the blue dot). Traditional estimation methods tend to favor high SNR signals (red dot) but have difficulty with low SNR activity with very high spatial correlations (blue dot). The EFD takes both spatial and temporal correlations into account and therefore detects both regions.

**Figure 11:**
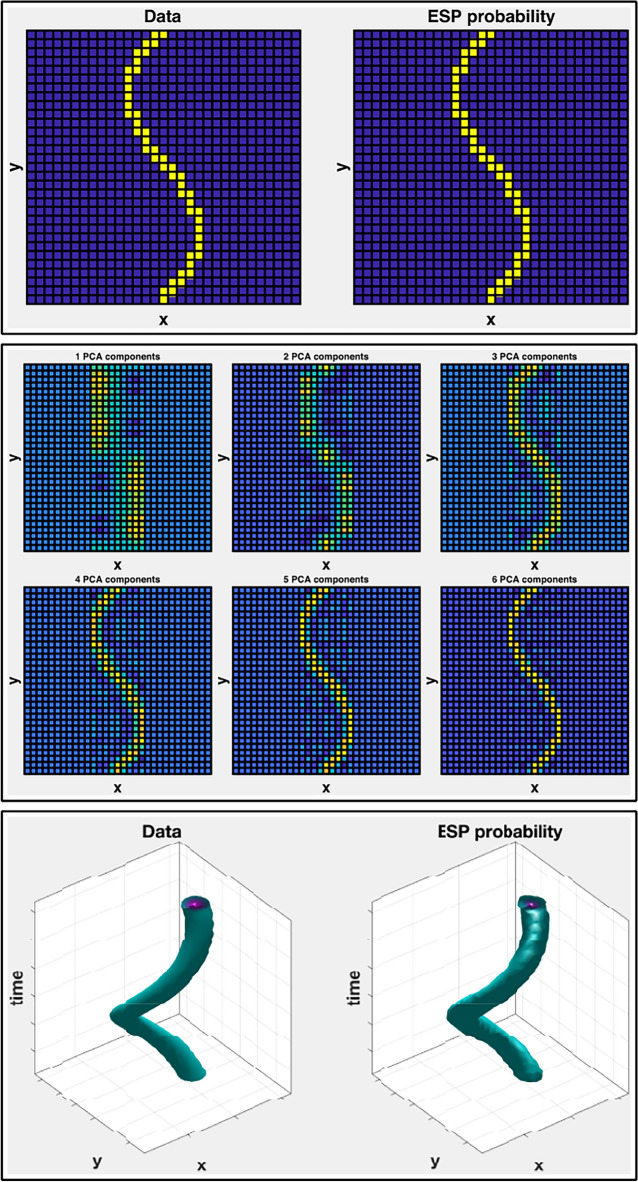
(Top left) Original lattice; (Top right) ESP probability. (Middle) Principal Components Analysis (PCA) results on same original lattice. The ESP probability locates the structure in a single calculation. The PCA decomposition even for 6 components shows significant errors. Several more components would be required to accurately fit the data. (Bottom row) Space-time ESP. (Top left) Space-time trajectory of 2D data; (Top right) ESP probability.

**Figure 12:**
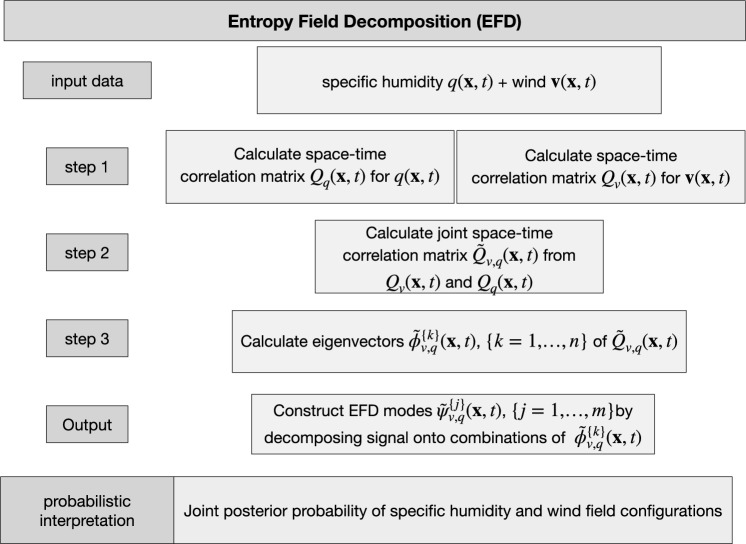
Schematic for the construction of space-time EFD modes from the data.

**Figure 13:**
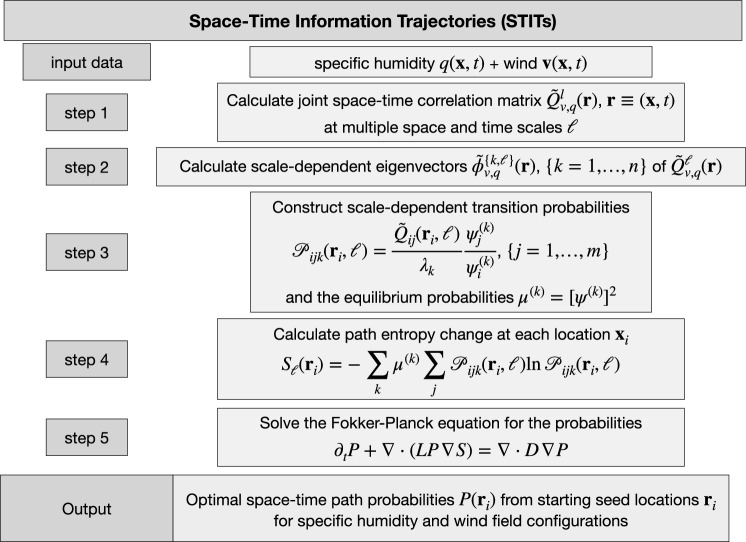
Schematic for the construction of space-time information trajectories (STITs) from the EFD modes constructed according to Fig. 12.

**Figure 14:**
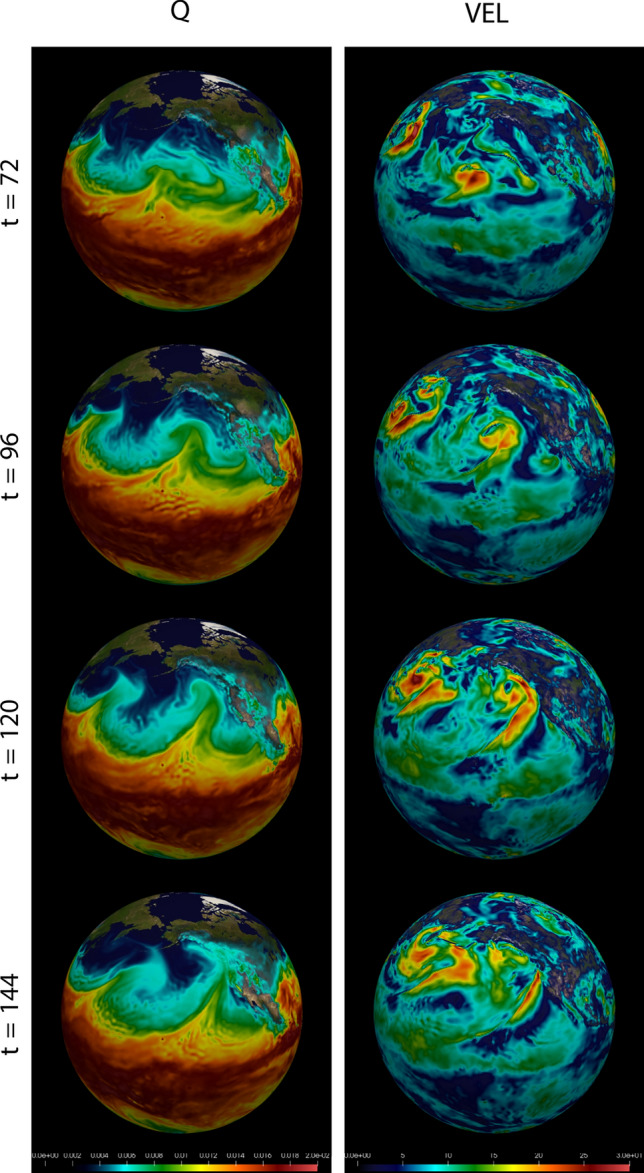
The wind (right column, $m s^{-1}$) and specific humidity (left column, *kg*/*kg*) fields at 850*hPa* the same time points shown for the EFD results for the Mar 2005 storm in Figure 2.

**Figure 15:**
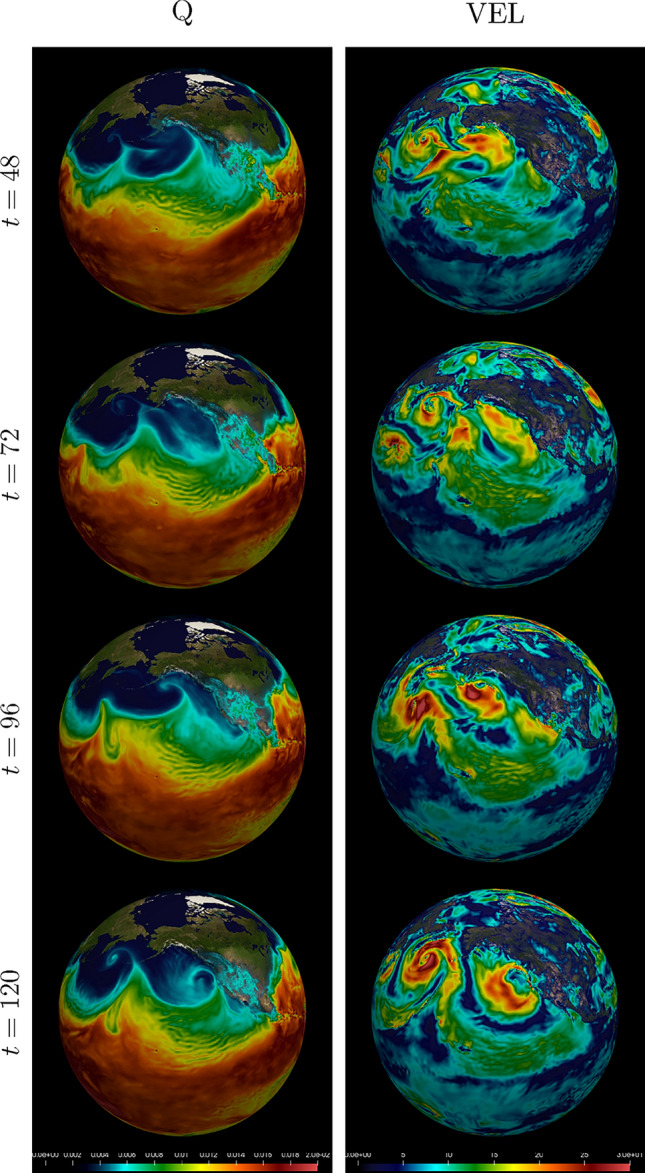
The wind (right column, $m s^{-1}$) and specific humidity (left column, *kg*/*kg*) fields at 850*hPa* the same time points shown for the EFD results for the Jan 2021 storm in Figure 3.

**Figure 16:**
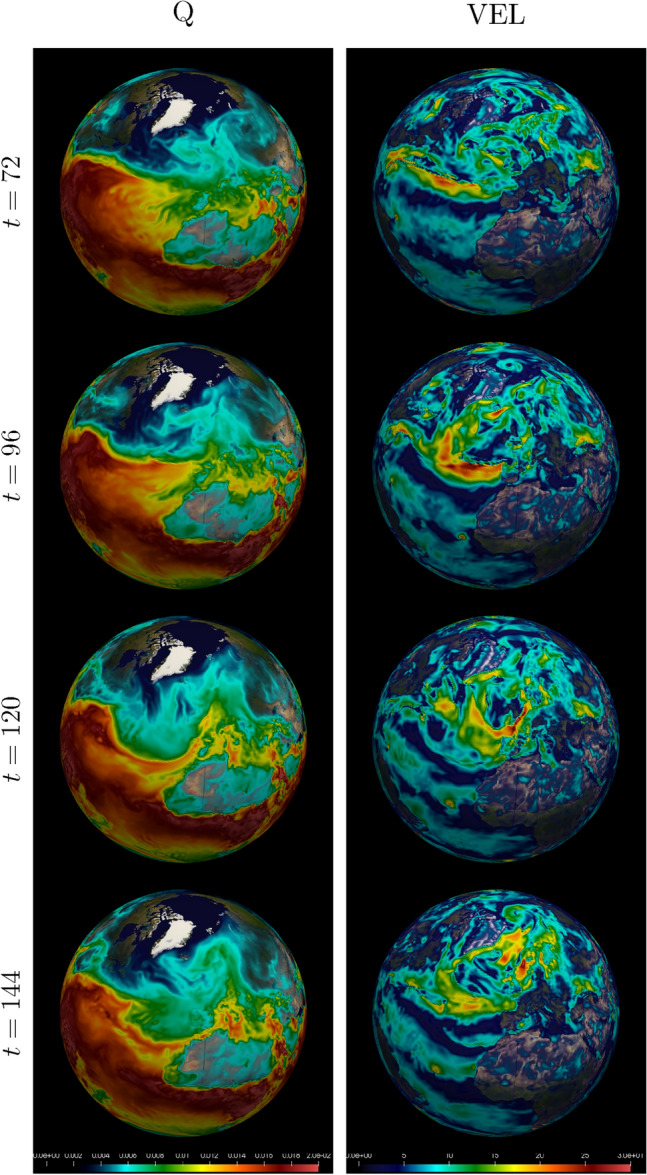
The wind (right column, $m s^{-1}$) and specific humidity (left column, *kg*/*kg*) fields at 850*hPa* the same time points shown for the EFD results for the Oct 1987 storm in Figure 5.

## Data Availability

The NCEP Climate Forecast System Reanalysis data (CFSR) used in this study is accessible from NCAR’s Research Data Archive https://rda.ucar.edu/datasets/ds093.0/ and Climate Forecast System Version 2 analysis data (CFSv2) is accessible from NCAR’s Research Data Archive https://rda.ucar.edu/datasets/ds094.0/. Underlying Earth satellite image in figures is in the public domain at https://commons.wikimedia.org/wiki/File:Land_shallow_topo_2048.jpg. Figures were made with Paraview version 5.10.0 (Kitware Inc., 2007) available under an open BSD license at https://www.paraview.org.

## A. Entropy field decomposition

### A.1 Inverse problems in meteorology

Analysis of weather systems can be considered from two viewpoints. One view is the theoretical construction of the equations governing atmospheric dynamics that when implemented computationally facilitates numerical exploration of the effects of different meteorological parameters on the final atmospheric configurations. This is the *forward problem*. The other view is attempting to reconstruct the actual weather system that was observed with real measured data. This is the *inverse problem*. These viewpoints are complementary, as the comparison of measurements with predicted results from theoretical models is the basis for the refinement of theoretical models of the atmosphere, while the incorporation of more refined models into the reconstruction problem can produce more accurate quantitative assessments of actual events.

However, exceedingly complex non-linear multivariate physical systems pose unique challenges, and the Earth’s weather is a prime example. It is a stratified multi-constituent, multi-phase fluid in an external (gravitational) field with an irregular, multi-phase, rotating lower boundary (the Earth’s surface). Consequently, atmospheric phenomena happen on a broad range of spatial and temporal scales, from planetary waves to severe local storms, and no theoretical construct can hope to precisely capture all the complexity of atmospheric dynamics. But even if this were possible, there is another significant confound: Non-linear systems are exquisitely sensitive to initial conditions in which small changes can result in vastly different final states. Indeed, it was in the study of weather where such “chaotic” systems were brought to widespread attention^41,42^ This means that even with well-defined governing equations the final state cannot be exactly predicted. Therefore the forward problem of simulating the weather from both idealized equations and unknown initial conditions will always be only an approximation, from an analytical perspective, and unpredictable with respect to initial conditions. Thus it is essential to have a general framework with which to address the inverse problem of characterizing atmospheric dynamics from *actual data* from meteorological measurements, which are the result of all the exact physical processes and initial conditions that actually occurred, however unknown.

However, solving inverse problems from data acquired from measurements of non-linear systems is a well-known challenge. Weather is particularly challenging because it varies over so many spatial and temporal scales and has so many interacting parameters (e.g., wind velocity, humidity, temperature, pressure, surface roughness, etc). This is in addition to the usual challenges of inverse problems, such as finite sampling and both instrument and environmental noise. Indeed, the analysis of dynamic non-linear systems poses a very general analysis problem across multiple scientific disciplines.

### A.2 Probabilistic approach

Characterizing the complex dynamics of a physical system by estimating the parameters of a hypothesized physical model falls under the purview of probability theory and was the motivation for our development of the entropy field decomposition (EFD), described in detail in^31,32^. Here we present a more intuitive overview (based on^31^), and limit the discussion to details pertinent to meteorological data.

The meteorological data we are interested in are 4-dimensional, sampled at 3 spatial dimensions and one time dimension. The spatial sampling is typically $\{ n_{lat},n_{lon},n_{lev} \}$ different latitudes, longitudes, and elevations, respectively. So the total number of points in a volume is $N = n_{lat} \times n_{lon} \times n_{lev}$. Measurements from this volume are made at a set of discrete times $t_{i}, i = 1, \ldots , n$ (assumed to be at equal intervals, though this is not a requirement of the analysis.) The data $\{ d_{i,j} \}$, $i=1,\ldots ,n$, , $j=1,\ldots ,N$ can equivalently be represented as $\{ d_l \}$, where ${\xi }_l, l=1,\ldots nN$ defines a set of space-time locations. Each data point is of the form

1 $$\begin{aligned} d_{i,j} = R s_{i,j} + e_{i,j} \end{aligned}$$

where *R* is an operator that represents the response of the measurement system to a signal $s_{i,j}$, and $e_{i,j}$ is the noise with the covariance matrix $\Sigma _e=\langle e e^\dagger \rangle$ where $\dagger$ means the complex conjugate transpose.

In NWP, the model equations are run forward in time using a set of initial conditions to generate the signal *s*, from which can be constructed data *d* by finite sampling the generated signal and adding noise and instrument constraints. For the inverse (or estimation) problem, the goal is to instead determine the signal *s* from the actual measured data *d*. This procedure is codified using Bayes Theorem to construct the posterior probability of the signal *s*, given the data *d*, and any information *I* that is known about the problem, including any assumptions, or hypotheses *H*:

2 $$\begin{aligned} \underbrace{p({s}|{d,I,H})}_{Posterior} = \frac{\overbrace{p({d}|{s, I, H})}^{Likelihood} \, \overbrace{p({s}|{I,H})}^{Prior}}{\underbrace{p({d}|{I,H})}_{Evidence}} \end{aligned}$$

The most probable signal is the one reconstructed from the configuration of estimated parameters to produce the maximum in this posterior distribution. The underlying signal is assumed to be continuous in space-time, and thus characterized by a field $\psi (x,t)\equiv \psi (\xi )$ although the data consist of discrete samples in both space and time so that the reconstructed underlying signal is $s_{l} = \int \psi ({\xi }) \delta ({\xi }-{\xi }_l) d{\xi }$.

While the Bayesian approach to signal analysis is not new (For an excellent introduction, see^58^), there have remained practical issues that have limited its applicability to non-linear interacting fields. By interacting we are referring to fields that influence one another. For example, in severe storms, the transport of moisture involves both the velocity field and the humidity field (among other things). The limitations in practical applications can be thought of as falling into two broad categories: (1) How to represent non-linear interacting fields and (2) How to incorporate relevant prior information. One approach to the first problem is reformulating Bayes Theorem in terms of field theory^59^, called *information field theory* (IFT), which facilitates the use of techniques developed in the physics discipline of field theory (e.g.,^60^). The advantage, and disadvantage, of this method is that it is essentially flexible in its ability to approximate any desired number of parameters and field interactions. This reformulation can be done by rewriting Bayes Theorem (Equation (2)) in the form^59^

3 $$\begin{aligned} p({\psi }|{d,I}) = \frac{e^{-H(d,\psi )}}{Z(d)} \end{aligned}$$

where the field theoretic quantities, the Hamiltonian $H(d,\psi )$ and the partition function *Z*(*d*), are

4a $$\begin{aligned} H(d,\psi )&= -\ln p({d,\psi }|{I}) \end{aligned}$$

4b $$\begin{aligned} Z(d) = p({d}|{I})&= \int \!\!d\psi \, e^{-H(d,\psi )} \end{aligned}$$

The partition function is a *generating function* from which can be constructed expressions for all orders of field interactions. For the current purposes, however, it will be considered a constant that can be ignored. The Hamiltonian describes the conserved quantities in field theories, and in IFT describes the conservation of probability. This will be the central quantity of interest. It is written

5 $$\begin{aligned} H(d,\psi ) = H_{0} - j^{\dagger } \psi + \frac{1}{2} \psi ^{\dagger } D^{-1} \psi + H_{i}(d,\psi ) \end{aligned}$$

where $H_{0}$ is essentially a normalizing constant that can be ignored, *D* is an information propagator, *j* is an information source, and $H_{i}$ is an interaction term (Eqn 7 in^31^). The solution for the fields is the minimum of the Hamiltonian ($\delta H/\delta \psi = 0$) is

6 $$\begin{aligned} \psi (\xi )&= D \left( j - H_{i}' \right) \end{aligned}$$

where $H_{i}'$ symbolically represent the variation if $H_{i}$ (the second term on the RHs of Eqn 8 in^31^).

The expressions Eqs. (5) and (6) are not in a form clearly equivalent to the more standard formulations of probability theory, so it is useful to demonstrate their equivalence in a simple problem. This will then make apparent where it deviates from standard formulations for use in more complex problems.

Consider the simple case in which the signal is assumed to be described by a set of model functions *F*, with each component weighted by an amplitude *a*. If the functions *F* do not interact with one another or the noise, the interaction term $H_{i}'$ in Eq. (6) can be ignored. Ignoring instrument effect ($R=1$) and assuming the signal is contaminated by zero mean Gaussian noise with variance $\Sigma _{e} = \sigma ^{2}$, the solution is^31^).

7 $$\begin{aligned} \psi (\xi ) = D j \quad \text{ where } \quad {\left\{ \begin{array}{ll} D = \sigma ^{2} (F^\dagger F)^{-1} \\ j= \sigma ^{-2} F^\dagger d \end{array}\right. } \end{aligned}$$

The source *j* is noise weighted projection of the signal onto the sampled model functions (sometimes called the “dirty map”^61^), and the propagator *D* characterizes the influence of the noise and the sampling of the functions *F* (sometimes called the “dirty beam”^61^). The estimated signal is

8 $$\begin{aligned} \psi (\xi ) = F^{+} d \quad \text{ where } \quad F^{+} = (F^\dagger F)^{-1} F^\dagger \end{aligned}$$

where $F^{+}$ is the well-known pseudo-inverse. Equation (8) is just the standard maximum *a posterior* result^62^. The rationale for the names ”source” and ”propagator” becomes clear. The input data $d(t_{i})$ projected along the *k*’th component of the model function $F(t_{i})$ provides the source *j* of new information, which is then propagated by *D* from which estimates of the field components are derived.

If the physical system were describable in terms of non-interacting plane waves, the *F* would be the standard Fourier functions, then the source is just the noise weighted inverse Fourier transform of the data while the propagator is the covariance of the sampled Fourier model functions. Each Fourier component would constitute a “mode” of the system, which would be a 4-dimensional (3 spatial and 1 temporal) time varying volume. These modes would be ranked according to their amplitude, or eigenvalue. This result is called the “Fourier decomposition” of the data and is ubiquitous across a wide range of scientific disciplines. It is important to note that this Bayesian analysis reveals that the estimate of the spatiotemporal patterns is *not* just the inverse Fourier transform of the data, but takes into account the uncertainties related to the sampling and the noise via the propagator *D*.

Unfortunately, many, perhaps most, physical systems are not easily characterized by such simple models. This is certainly the case for the planetary weather systems considered in this paper, where a more appropriate general model is that of non-linear, non-periodic, interacting fields. This makes the process of estimating the system from the data much more difficult, because many more combinations of the model parameters are consistent with the data. In addition, non-linear systems can be exquisitely sensitive to initial conditions, which are rarely known precisely and again can produce a wide range of results for the same set of system parameters. The IFT provides a framework to formally characterize complex interacting non-linear fields, but no guidance on resolving these ambiguities. The resulting field theoretic description produces expressions with an essentially infinite number of terms. Without a method for effectively ranking the relative importance of the terms, problems can quickly become intractable.

### A.3 The role of prior information

Solving seemingly intractable inverse problems requires the reduction in the number of possible system configurations (i.e., the set of parameters characterizing the system) that are consistent with the data. This can be achieved through the development of increasingly refined theoretical physical models. But probability theory provides another powerful mechanism, which is the ability to incorporate prior information. Formally, this is done through the “prior” in Eq. (2). However, the apparent simplicity of this term belies the complexity in using it. One common and conceptually simple use is to incorporate statistical parameters from previous measurements that characterize a distribution known, or estimated, to describe the system. For example, for a system whose parameters are assumed to be normally distributed, estimates of average and standard deviation can be used to construct a Gaussian prior distribution. While such an approach is common, it is not particular useful when systems are highly non-linear and non-Gaussian.

One of the remarkable traits of the Earth’s atmospheric dynamics is that, despite its seemingly chaotic nature, it displays the remarkable ability to produce phenomena that are highly coherent in space and time, such as planetary waves, hurricanes, and tornadoes. Characterizing such coherences is one of the major objectives of meteorological data analysis. Observations of such data bring up a subtle but important issue that arises in the estimation of coherent space-time processes. By coherence we mean that there are spatial patterns that are persistent in time. For example, a tornado is a persistent spatially localized vorticity. While these notions are intuitively clear, they are notoriously difficult to formalize in analytical theories of estimation. Consider for example the idealized numerical simulation shown in Fig. 10 in which there are two areas of activity in Gaussian random noise: a point oscillating with very high signal-to-noise (SNR) (centered on the red dot) but also a larger circular region with very low SNR (centered at the blue dot). The computation question can be phrased in terms of “What is the significance of the activated regions?” In more intuitive terms, while the high SNR region clearly seems to indicate an area of activity, we intuitively understand that the larger region is significant despite its low amplitude because it is so persistently coherent over such a large spatial region. Is there a way to incorporate the spatial coherence into the computation of significance?

### A.4 Entropy spectrum pathways (ESP)

The problem then is this: How does one identify regions of spatial and temporal coherence in a dataset that contains complex spatiotemporal patterns that are unique from event to event and thus not amenable to fitting by some standard model? This was the problem faced by Lorenz who recognized that the implications of the unpredictability of atmospheric systems for estimation theory required a general method that could be applied to any dataset from such systems, which led him to the formulation of *empirical orthogonal functions* (**EOF**)^44^, which is synonymous with the *principal components analysis* (**PCA**)^63^. On the face of it, PCA is “model free” in that the physical model is not parameterized. However, it is based on the assumption that the data can be described by a multivariate Gaussian distribution and proceeds by successively fitting the data to *n* ellipsoids following the subtraction of the previous $n-1$ components. While PCA is easy to implement, it typically is a poor model for complex physical systems and the resulting components therefore can often have little relationship to the actual coherent modes within the data, and therefore makes identification of physically relevant parameters more problematic.

Remarkably, one answer to this question of how to incorporate prior information on system coherences is hidden in plain sight—it is contained in the data itself. Spatial and temporal coherences are characterized by being significantly correlated with their neighbors. In an image, this could simply be the similarity of intensities in neighboring spatial locations. In meteorological data, it might take the form of similar velocity vectors in neighboring regions of both space and time. This concept can be formalized by computing space-time correlations from the data, which are local interactions (i.e., computed in adjacent spatial and temporal points in the data) but, when computed at every location in a dataset, also provide information about the larger scale coherent structures in the data. The relationship between these local interaction and the large scale structures in the data is subject of the theory of *entropy spectrum pathways* (**ESP**)^46^.

The essence of the theory is that the coupling between adjacent (in space or time) data elements *i* and *j* can be viewed as a measure of the information that can be transmitted between those two locations. One could imagine computing the correlation between the copper content in adjacent elements of a volumetric image of a transmission cable. The resulting high probability regions of adjacent high correlations would be the copper wire, and therefore literally be the path of maximum information transmission. This concept can be formalized and abstracted to any dataset using and parameter or set of parameters. The local interactions reveal the large scale pathways in the data and therefore the pathways of information transmission about the coherences of these parameters. This concept is formalized by computing a *coupling matrix* $Q_{ij}$ between adjacent data elements *i* and *j* and computing its eigenvectors $\phi ^{(k)}$, $k=1,\ldots ,M$. (For a detailed discussion of the construction of $Q_{ij}$, see^31^.) Remarkably, these eigenvectors can be interpreted as the pathways between locations that can be reached in the most number of ways. In other words, the path in the data that have maximum path entropy. In the wire example, an electron starting at any location on the wire would preferentially travel along that wire. The eigenvector with the largest eigenvalue ($k=1$), the principal eigenvector, defines the maximum entropy pathway, that which is most preferred.

A simple example of ESP to uncover spatial correlations is shown in Fig. 11 (Top) where a simple 2D spatial curve is detected with the principal eigenvector of the coupling matrix formed from the intensities at each location. This is to be compared with the standard PCA result in Fig. 11 (Middle) that required multiple components to be computed and still only approximately detects the correct structure. The extension to space-time is shown in Fig. 11 (Bottom) where the principal ESP eigenvector correctly picks out the preferred space-time pathway. PCA in this case (not shown) becomes even more problematic.

The recognition that non-Gaussian systems are poorly described by PCA leads to the formulation of a non-linear version called *independent components analysis* (**ICA**) which seeks to decompose data into independent non-Gaussian components. However, this “model free” method has also contains hidden assumptions that produce unreliable results in non-linear complex physical systems (see^32^ for a more extended discussion and examples).

### A.5 The entropy field decomposition

The ESP prior can be incorporated into the estimation scheme by using the coupling matrix $Q_{ij}$ as the prior in Equation (2)

9 $$\begin{aligned} p({s}|{d,I}) = \frac{1}{|{2\pi Q}|^{1/2}} \exp \left( -\frac{1}{2} {s}^{\dagger }_{i} Q_{ij} {s}^{\phantom {\dagger }}_{j} \right) \end{aligned}$$

which then becomes part of the Hamiltonian in Equation (3). (For a detailed discussion of the construction of $Q_{ij}$, see^31^.) The result is that the significant spatial and temporal correlations within the data can provide highly relevant prior information about the most probable parameters configurations of a physical system, and select out only the limited number of relevant terms within the infinite number of choices that make the inverse problem consistent with the data. The incorporation of the ESP theory into the Bayesian framework of IFT is called the *entropy field decomposition* (**EFD**).

Returning to the simple example above, with the ESP prior, the solution given in Eq. (7) is the same but with a modified propagator that now includes the coupling matrix:

10 $$\begin{aligned} D=\left[ Q+F^\dagger \Sigma _{e}^{-1} F\right] ^{-1} \end{aligned}$$

This means that the estimated modes of the system are now dependent on the coupling matrix *Q*. And in particular, the optimal solutions depend on the eigenvectors $\phi$ of *Q*. Therefore the EFD modes are found by expressing the probability, that is, the Hamiltonian, in terms of $\phi$. In other words, whereas in the simplest, uncoupled problem above the Fourier functions serve as the proper ’basis’ to describe the problem, now the eigenfunction of the *Q* serves that function. These eigenvectors incorporate both the spatial and the temporal correlations, and thus address the problem shown in Fig. 10: the significance of the detected activated region is enhanced by the adjacency, or clustering, of the activated voxels, as we intuitively believe. Therefore, no post-hoc clustering of voxels determined independently to be activated based on their time course is required, which is the current widespread methodology for detecting activation in, for example, functional MRI (fMRI) data^64–67^.

This extends to the more general solution where interactions between fields are considered as well and the interaction Hamiltonian $H_{i}'$ (Eq. (6)) is not ignored (see^31^ for details). In general, solving the eigenvalue problem for *Q* produces an *transition probability* $p_{ijk}$ between locations (*i*, *j*) for the *k*’th mode and an *equilibrium distribution* $\mu ^{(k)}$ associated with the set of $k=1,M$ mode eigenvectors $\phi ^{(k)}$. The EFD procedure produces *M* modes that are ranked by their eigenvalues, providing a simple, unsupervised method for characterizing the primary modes of data collected from an arbitrarily complex non-linear, non-periodic, non-Gaussian physical system.

A compelling feature of the EFD approach is that the basis functions *are unique to every problem*, since they are derived directly from the data. This makes the EFD approach powerful for physical systems that are always unique, such as weather systems. Moreover, the key practical issue is that the coupling matrix *Q* can be defined by the user according to the problem at hand. In severe weather Doppler radar data, for example, the amplitude of dBZ can be used to map large scale storm structure (e.g.,^33^ Fig. 8), or the vertical vorticity for more localized dynamics of tornado formation (e.g.,^33^ Fig. 10). A useful demonstration of what is meant by “modes” is demonstrated by a simple toy problem of two helical Burgers vortex tubes shown in^33^, Fig. 1. In the current paper, we are interested in both the specific humidity *q* and wind field $\varvec{v}$. The details of incorporating multiple parameters into the coupling matrix are discussed in^45^. A schematic of the EFD procedure is shown in Fig. 12.

### A.6 Multi-modality EFD (JESTER)

Extending the EFD methods to multiple modalities by incorporating coupling between different parameters, which we call *Joint Estimation with Entropy Regularization* (JESTER)^45^, is accomplished as follows. For $m=1,...,M$ different modalities $d^{(m)}$ with the coupling matrices $Q^{(m)}$ that all correspond to the same unknown signal *s*, intermodality coupling matrix can be constructed as the product of the coupling matrices for the individual modalities expressed in the ESP basis and registered to a common reference frame, which we denote ${\widetilde{Q}}^{(m)}$: That is, the joint coupling matrix is $\varvec{\mathcal {Q}}^{(m)} = \prod _{m} {\widetilde{Q}}^{(m)}$. More specifically, the joint coupling matrix $\varvec{\mathcal {Q}}_{ij}$ between any two space-time locations (*i*, *j*) can be written in the general (equivalent) form as

11 $$\begin{aligned} \ln \varvec{\mathcal {Q}}_{ij} = \sum _{m=1}^M \beta _{ij}^{(m)} \ln {\widetilde{Q}}_{i j}^{(m)} \end{aligned}$$

where the exponents $\beta ^{(m)}$ can either be some constants or functions of data collected for different modalities $\beta _{ij}^{(m)}\equiv \beta ^{(m)} (\varvec{{\widetilde{d}}_{i}} ,\varvec{{\widetilde{d}}_{j}} ), \varvec{{\widetilde{d}}_{i}} \equiv \{ {\widetilde{d}}_{i}^{(1)} ,..., {\widetilde{d}}_{i}^{(M)} \}$ where ${\widetilde{d}}_{i}^{(m)}$ and ${\widetilde{Q}}_{ij}^{(m)}$ represent, respectively, the data and the coupling matrix of the modality dataset *m* represented in the ESP basis and evaluated at locations $r_i$ and $r_j$ of a common reference domain *R*:

12 $$\begin{aligned} {\widetilde{d}}_i^{(m)} = {d}^{(m)} \left( \psi ^{(m)}(r_i)\right) , \qquad {\widetilde{Q}}_{ij}^{(m)} = Q^{(m)} \left( \psi ^{(m)}(r_i),\psi ^{(m)}(r_j)\right) \end{aligned}$$

where $\psi ^{(m)}:R \rightarrow X$ denotes a diffeomorphic mapping of *m*-th modality from the reference domain *R* to an acquisition space *X*.

### A.7 Space-time information trajectories

While very spatially localized estimation of ARs produced by the EFD modes is of great operational significance, it does not lend much insight into the influences of the larger scale structure of the synoptic and planetary atmospheric dynamics that cause them. The importance of discerning such influences is well recognized and motivated the development of ’tracking’ method typically based on standard Lagrangian methodologies (e.g.,^68^)

The EFD framework provides a powerful method for characterizing the space-time structure of physical systems from observational data. The estimation of the EFD modes is based on the spatiotemporal correlations of the data, from which the most probable configuration of the parameters determines the space-time modes. But it is also possible to consider the spatiotemporal evolution of the highest probability pathways, those of maximum correlation.

Calculation of the ESP modes produces the equilibrium $\mu ^{(k)}$ distributions and transitional probabilities $p_{ijk}$ were obtained from the space-time nearest neighbor coupling matrix $Q_{ij}^{k}(x_{i},x_{j},t)$. (For a detailed discussion of the construction of $Q_{ij}$, see^31^.) These can be used to construct space-time information trajectories defined as streamlines generated using the global path entropy change

13 $$\begin{aligned} S_{i} = -\sum _{k}{\mu }^{(k)}\sum _{j} p_{ijk} \ln p_{ijk} \end{aligned}$$

The global entropy field Eq. (13) was obtained under the Markovian assumption in the limit of long pathway lengths (or large times)^69,70^ and describes the global flow of information. The trajectories are generated by linearizing the Fokker–Planck equation with a potential in the form of the global entropy Eq. (13)^46^ and finding the characteristics solution using the Hamiltonian set of equations to construct the space-time trajectories are generated using a geometric-optics based approach, as described in^71^. Multiple *connectivity eigenmodes* (**CEM**) generated from each STIT by computing the eigenvectors of the connectivity matrix between all points $x_{i}$ and $x_{j}$ characterize the primary information pathways in the data. For more details, see^32^, and for their use in meteorological data see^33^. A schematic of the STIT procedure is shown in Fig. 13.

## B Wind and specific humidity data

The general EFD method is capable of analyzing fields (e.g., wind, humidity, pressure, etc.) either individually or jointing. In the present paper, the focus on ARs motivated the joint analysis of humidity and wind fields. In this Appendix we show the raw input humidity and wind data (the latter vector field in terms of the scalar velocity) that were used as input into the EFD algorithm.

The wind and specific humidity fields at the same time points discussed in the EFD results in "Results" section are shown in for Jan 2021, Mar 2005, and Oct 1987 events in Figs. 14, 15, 16 respectively.

## C Computational implementation

All the algorithms implemented in this paper were written in standard ANSI C/C++ and parallelized using POSIX threads, resulting in an efficient computational implementation. The computations in the paper of three EFD modes $\psi ^{(0,1,2)}$ of wind coupled with specific humidity on the 3.1GB data of size $\left( nlon,nlat,nlev,nt \right) = \left( 720,361,19,40 \right)$ took $\approx 28$ min on a Dual E5-2697 v3 2.6 GHz Fourteen-Core 145W Linux machine with 512GB DDR4 EEC Registered Memory. STIT and subsequent CEM computations for each mode took approximately 23 min.
